# A human functional protein interaction network and its application to cancer data analysis

**DOI:** 10.1186/gb-2010-11-5-r53

**Published:** 2010-05-19

**Authors:** Guanming Wu, Xin Feng, Lincoln Stein

**Affiliations:** 1 Ontario Institute for Cancer Research, MaRS Centre, South Tower, 101 College Street, Suite 800, Toronto, ON M5G 0A3, Canada; 2 Cold Spring Harbor Laboratory, One Bungtown Road, Cold Spring Harbor, NY 11724, USA; 3 Stony Brook University, Stony Brook, NY 11794, USA

## Abstract

**Background:**

One challenge facing biologists is to tease out useful information from massive data sets for further analysis. A pathway-based analysis may shed light by projecting candidate genes onto protein functional relationship networks. We are building such a pathway-based analysis system.

**Results:**

We have constructed a protein functional interaction network by extending curated pathways with non-curated sources of information, including protein-protein interactions, gene coexpression, protein domain interaction, Gene Ontology (GO) annotations and text-mined protein interactions, which cover close to 50% of the human proteome. By applying this network to two glioblastoma multiforme (GBM) data sets and projecting cancer candidate genes onto the network, we found that the majority of GBM candidate genes form a cluster and are closer than expected by chance, and the majority of GBM samples have sequence-altered genes in two network modules, one mainly comprising genes whose products are localized in the cytoplasm and plasma membrane, and another comprising gene products in the nucleus. Both modules are highly enriched in known oncogenes, tumor suppressors and genes involved in signal transduction. Similar network patterns were also found in breast, colorectal and pancreatic cancers.

**Conclusions:**

We have built a highly reliable functional interaction network upon expert-curated pathways and applied this network to the analysis of two genome-wide GBM and several other cancer data sets. The network patterns revealed from our results suggest common mechanisms in the cancer biology. Our system should provide a foundation for a network or pathway-based analysis platform for cancer and other diseases.

## Abstract 2

A high-quality human functional protein interaction network is constructed. Its utility is demonstrated in the identification of cancer candidate genes.

## Background

High-throughput functional experiments, including genetic linkage/association studies, examinations of copy number variants in somatic and germline cells, and microarray expression experiments, typically generate multiple candidate genes, ranging from a handful to several thousands. These data sets are noisy and contain false positives in addition to genes that are truly involved in the biological process under study. An unsolved challenge is how to understand the functional significance of multi-gene data sets, extract true positive candidate genes, and tease out functional relationships among these genes with confidence for use in further experimental analysis.

### Using biological pathways to interpret high-throughput data

One way to approach the above problem is to analyze the data from the perspective of biological pathways [1,2]. A pathway is a set of biochemical events that drives a cellular process. For example, the transforming growth factor beta (TGFβ) pathway consists of a ligand receptor binding event that initiates a series of protein-protein interaction (PPI), protein degradation, protein phosphorylation, and protein-DNA binding events that transmit a regulatory signal and regulate proliferation, differentiation and migration [3]. In cancer, the TGFβ signaling network functions in complex ways to both suppress early tumor growth and promote late stage progression [4]. Some breast cancers [5-9] are thought to arise in part when components of the TGFβ pathway are deleted, thereby freeing the tissue from growth inhibition. The same type of cancer can arise via several different routes [2]. For example, tumors from two different patients might have deleted different components of the TGFβ pathway. Although the two tumors both share the loss of TGFβ growth inhibition, they may not share defects in a common gene or gene sets. However, a pathway-based analysis will resolve this confusing finding and point towards the etiology of the disease. By projecting the list of mutated, amplified or deleted genes onto biological pathways, one will find that a statistically unlikely subset of otherwise unrelated genes are closely clustered in 'reaction space'. Pathway-based analysis can thus provide important insights into the biology underlying disease etiology. One striking example of this approach is the finding of the 'exclusivity principle' in cancer: only one gene is generally mutated in one pathway in any single tumor [1].

Recently, several large-scale genome-wide screening projects have revealed common core signaling pathways in the etiology or progression of several cancer types [10-14], indicating the relevance of pathway-based analysis for the understanding of large scale disease data sets. Pathway-based analysis accomplishes at least two things: it marks the genes associated with the disease or other phenotype and separates them from innocent bystanders caught in the general instability of the malignant genome or other false positive hits [15]; and it identifies the biological pathways affected by the genes [16]. The latter outcome also places the high-throughput analysis results in an intellectual framework that can be more easily comprehended by the researcher. It connects his results to prior work from the literature, and allows him to propose hypotheses that can be tested by further experimental work.

### Resources for pathway analysis

Pathway-based hypothesis generation has been the subject of great interest over the past few years [17]. It is the basis for several popular data analysis systems, including GOMiner [18,19], Gene Set Enrichment Analysis [20], Eu.Gene Analyzer [21], and several commercial tools (for example, Ingenuity Systems [22]).

Reactome [23] is an expert-curated, highly reliable knowledgebase of human biological pathways. Pathways in Reactome are described as a series of molecular events that transform one or more input physical entities into one or more output entities in catalyzed or regulated ways by other entities. Entities include small molecules, proteins, complexes, post-translationally modified proteins, and nucleic acid sequences. Each physical entity, whether it be a small molecule, a protein or a nucleic acid, is assigned a unique accession number and associated with a stable online database. This connects curated data in Reactome with online repositories of genome-scale data such as UniProt [24] and EntrezGenes [25], and makes it possible to unambiguously associate a position on the genome with a component of a pathway. A computable data model and highly reliable data sets make Reactome an ideal platform for a pathway-based data analysis system. However, since all data in Reactome is expert-curated and peer-reviewed to ensure high quality, the usage of Reactome as a platform for high-throughput data analysis suffers from a low coverage of human proteins. As of release 29 (June 2009), Reactome contains 4,181 human proteins, roughly 20% of total SwissProt proteins. Other curated pathway databases, including KEGG [26], Panther Pathways [27], and INOH [28], offer similarly low coverage of the genome.

In contrast to pathway databases, collections of pairwise relationships among proteins and genes offer much higher coverage. These include data sets of PPIs and gene co-expression derived from multiple high-throughput techniques such as yeast two-hybrid techniques, mass spectrometry pull down experiments, and DNA microarrays. These kinds of data sets are readily available from many public databases. For example, PPIs can be downloaded from BioGrid [29], the Database of Interacting Proteins [30], the Human Protein Reference Database (HPRD) [31], I2D [32], IntACT [33], and MINT [34], and expression data sets from the Stanford Microarray Database [35] and the Gene Expression Omnibus [36]. Protein or gene networks based on these pairwise relationships have been widely used in cancer and other disease data analysis with promising results [37-42].

### Transforming pairwise interactions into probable functional interactions

A limitation of pairwise networks is that the presence of an interaction between two genes or proteins does not necessarily indicate a biologically functional relationship; for example, two proteins may physically interact in a yeast two-hybrid experiment without this signifying that such an interaction forms a part of a biologically meaningful pathway in the living organism. In addition, some pairwise interaction data sets may have high false positive rates [43,44], which contribute noise to the system, and interfere with pathway-based analyses. For this reason, groups that make pathway-based inferences on high-throughput functional data sets inevitably draw on curated pathway projects to cleanse their data and to train their predictive models.

Our goal is to achieve the best of both worlds by combining high-coverage, unreliable pairwise data sets with low-coverage, highly reliable pathways to create a pathway-informed data analysis system for high-throughput data analysis. As the first step towards achieving this goal, we have created a functional interaction (FI) network that combines curated interactions from Reactome and other pathway databases, with uncurated pairwise relationships gleaned from physical PPIs in human and model organisms, gene co-expression data, protein domain-domain interactions, protein interactions generated from text mining, and GO annotations. Our approach uses a naïve Bayes classifier (NBC) to distinguish high-likelihood FIs from non-functional pairwise relationships as well as outright false positives.

**Figure 1:**
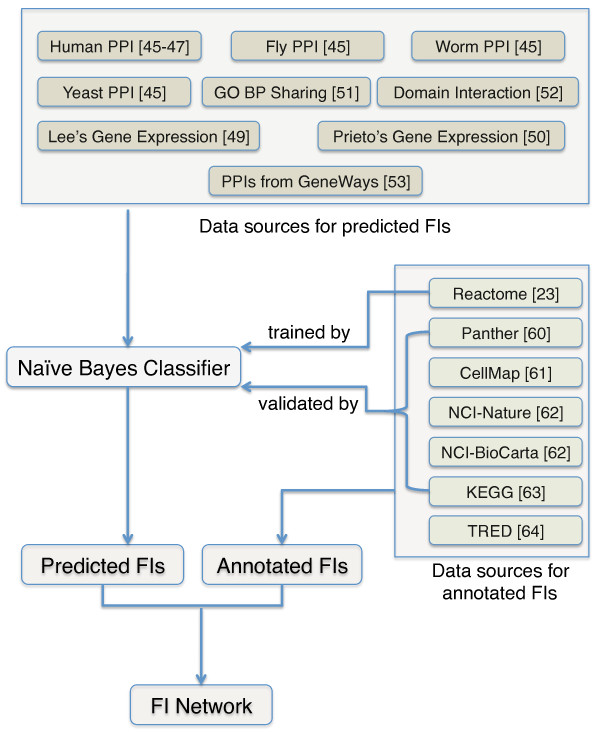
**Overview of procedures used to construct the functional interaction network**. See text for details. BP, biological process.

In this report, we describe the procedures to construct this FI network (Figure 1), and apply this network to the study of glioblastoma multiforme (GBM) and other cancer types by expanding a human curated GBM pathway using our FIs, projecting cancer candidate genes onto the FI network to reveal the patterns of the distribution of these genes in the network, and utilizing network clustering results on cancer samples to search for common mechanisms among many samples with different sequence-altered genes. Finally, we introduce a web-based user interface that gives researchers interactive access to the derived FIs.

## Results

### Data sources used to predict protein functional interactions

We used the following six classes of data to predict protein FIs (Table 1): 1, human physical PPIs catalogued in IntAct [45], HPRD [46], and BioGrid [47]; 2, human PPIs projected from fly, worm and yeast in IntAct [45] based on Ensembl Compara [48]; 3, human gene co-expression derived from DNA microarray studies (two data sets [49,50]); 4, shared GO biological process annotations [51]; 5, protein domain-domain interactions from PFam [52]; and 6, PPIs extracted from the biomedical literature by the text-mining engine GeneWays [53].

**Table 1:** Data sources used to predict protein functional interactions

Data source	Proteins	SwissProt proteins (coverage)	Interactions	Reference
Human PPIs	10,287	10,029 (49%)	53,743	[45-47]
Fly PPIs	13,383	4,088 (20%)	939,639 (26,346^a^)	[45]
Worm PPIs	5,223	1,477 (7%)	122,192 (8,161^a^)	[45]
Yeast PPIs	5,646	1,530 (8%)	1,900,980 (167,574^a^)	[45]
Domain interaction	60,569	15,218 (75%)	NA	[52]
Lee's Gene Expression	8,250	7,647 (38%)	206,117	[49]
Prieto's Gene Expression	3,024	2,901 (14%)	13,441	[50]
GO BP sharing	14,197	14,197 (70%)	NA	[51]
PPIs from GeneWays	5,252	5,252(26%)	51,048	[53]

To calculate the coverage of SwissProt, we used 20,332, the total identifier number in SwissProt (UniProtKB/Swiss-Prot Release 56.9, 3 March 2009), as the denominator. The numbers of interactions from three model organisms have been mapped to human proteins based on Ensembl Compara [48] (see text for details). ^a^Numbers of PPIs in the original species. BP, biological process.

Table 1 lists these data sources, the numbers of proteins and interactions, and estimated coverage of the human genome expressed as their coverage of the SwissProt protein database.

The coverage ranges from 7% (Worm PPIs) to 70% (GO biological process sharing). It is notable that the coverage of human physical PPIs from three public protein interaction databases (IntAct, HPRD, and BioGrid) is close to 50%. Many interactions from IntAct were catalogued from co-immunoprecipitation experiments combined with mass spectrometry, and contain multiple proteins in a single interaction record. An odds ratio analysis showed that human PPIs based on all interaction records are much less correlated to FIs (see below) extracted from Reactome pathways than interactions containing four or fewer interactors: 13.91 ± 0.52 versus 36.98 ± 9.17 (*P*-value = 2.8 × 10^-5 ^based on *t*-test). Therefore, we selected interactions that contain only four or fewer interactors from the IntAct database. We also tried to use GO molecular functional annotations as one of the data sources. The odds ratio of this data set was 2.99 ± 0.02, much smaller than the GO biological process data set (11.85 ± 0.20). Our results show that this data set contributed little to the prediction. One reason for this may be that the GO molecular functional categories are usually broad and the purpose of our NBC is to predict if two proteins may be involved in the same specific reactions (see below).

### Construction and training of a functional interaction classifier

Our goal was to create a network of protein functional relationships that reflect functionally significant molecular events in cellular pathways. The majority of PPIs in interaction databases are catalogued as physical interactions, and there is rarely direct evidence in the interaction databases that these interactions are involved in biochemical events that occur in the living cell. Other protein pairwise relationships have similar issues. To integrate pairwise relationships into a pathway context, we built a scoring system based on the NBC algorithm, a simple machine learning technique [54], to score the probability that a protein pairwise relationship reflects a functional pathway event.

For our NBC, we used nine features as listed under 'Data source' in Table 1: 1, whether there is a reported PPI between the human proteins; 2, whether there is a reported PPI between the fly (*Drosophila melanogaster*) orthologs of the two human proteins; 3, whether there is a reported PPI between the worm (*Caenorhabditis elegans*) orthologs of the two human proteins; 4, whether there is a reported PPI between the yeast (*Saccharomyces cerevesiae*) orthologs of the two human proteins; 5, whether there is a domain-domain interaction between the human proteins; 6 and 7, whether the genes encoding the two proteins are co-expressed in expression microarrays based on two independent DNA array data sets; 8, whether the GO biological process annotations for human proteins are shared; and 9, whether there is a text-mined interaction between the human proteins.

An NBC must be trained using positive and negative training data sets in order to determine the proper weighting of different combinations of features. We developed training sets from the curated information in Reactome, relying in part on an independent analysis that reported Reactome as a highly accurate data set for PPI prediction [55].

An issue in using PPIs and other pairwise relationships in a pathway context is that the data models used by pathway databases are much richer than a simple binary relationship. A pathway database describes pathways in terms of proteins, small molecules and cellular compartments that are related by biochemical reactions that have inputs, outputs, catalysts, cofactors and other regulatory molecules. To develop the training sets from Reactome pathways for NBCs, we established a relationship called 'functional interaction' using the following definition: a functional interaction is one in which two proteins are involved in the same biochemical reaction as an input, catalyst, activator, or inhibitor, or as two members of the same protein complex.

It is important to note that in Reactome a 'reaction' is a general term used to describe any discrete event in a biological process, including biochemical reactions, binding interactions, macromolecule complex assembly, transport reactions, conformational changes, and post-translational modifications [23]. We treat two members of the same protein complex as functionally interacting with each other because the activity of the complex as a whole is presumably functionally dependent on the presence of all of its subunits.

Based on the above definition, we extracted 74,869 FIs from Reactome, and used these FIs to create a positive training set for the NBC. After filtering out FIs that did not have at least one feature derived from the data sources in Table 1, the positive data set comprised 45,079 FIs.

Creating a good negative training set is more difficult than creating a positive set due to the incompleteness of our knowledge of protein interactions [56]: just because two proteins are not known to interact does not mean that this does not in fact occur. Research groups have addressed this problem using a variety of approaches, including choosing protein pairs from different disjunct cell compartments [57], or random pairs from all proteins [58]. For our NBC training, we followed the method in Zhang *et al. *[58] using random pairs selected from proteins in the filtered Reactome FI set.

Choosing an appropriate prior probability or ratio between the positive and negative data sets is important for NBC training. We calculated the prior probability based on the total number of proteins in the filtered FIs from Reactome pathways, which was 5.7 × 10^-3^. To check the effect of ratio between the sizes of the positive and negative data sets, we test the NBC performance using a ratio of either 10 or 100. NBCs trained with these two ratios yielded similar true and false positive rates, which indicated that our NBC is robust against the size of the negative data set.

**Figure 2:**
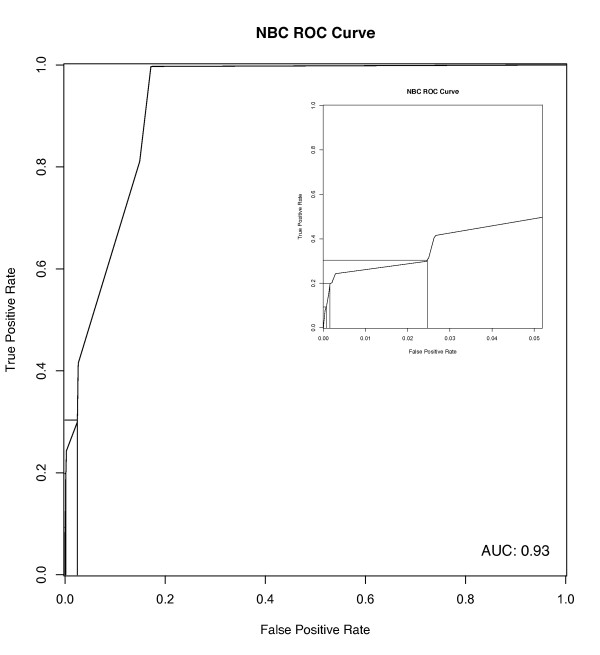
**Receiver operating characteristic curve for NBC trained with protein pairs extracted from Reactome pathways as the positive data set, and random pairs as the negative data set**. This curve was created using an independent test data set generated from pathways imported from non-Reactome pathway databases. The positions for the cutoff values 0.25, 0.50 and 0.75 are marked from right to left in the inset. The area under the curve (AUC) for this receiver operating characteristic (ROC) curve is 0.93.

The performance of machine learning classifier systems can be evaluated by cross-validation, or more stringently by using an independent data set. We used FIs extracted from pathways in other human curated pathway databases as a testing data set to evaluate the performance of our trained NBC. Figure 2 shows a receiver operating characteristic curve that relates true positive rates to false positive rates across a range of thresholds using this testing data set. We chose a threshold score of 0.50, which trades off a high specificity of 99.8% against a low sensitivity of 20%. The low sensitivity may result, in part, from high false negative rates existing in some of the data sets we used for NBC, especially in PPIs [59].

At the threshold score (0.50), a protein pair must have multiple types of FI evidence in order to be scored as a true FI (Table S1 in Additional file 1). While most (97%) of the predicted FIs have at least one PPI feature (Figure S1 in Additional file 1), there are no predictions supported solely by human PPI data, and fewer than 3% are supported solely by PPIs in human plus other species. This greatly reduces the weight given to raw human PPI features: the 44,819 human PPIs that went in to the classifier as features resulted in fewer than 15,000 predicted FIs, representing the removal of 68% of the raw PPIs. Most (75%) of the predicted FIs are derived from GO biological process term sharing and protein domain interactions in addition to PPIs.

As a check on the classifier's ability to enrich for FIs, we compared the sharing of GO cellular component annotations (which includes compartments such as 'nucleoplasm') among raw human PPIs to the sharing of these annotations among predicted FIs. Since GO cellular component annotations were not used as a feature during NBC training, we reasoned that this assessment should be independent. Among raw PPIs, 62.9% share GO cellular component terms annotated for both proteins involved in the interaction. In contrast, 96.2% of the predicted FIs share this type of GO term (*P*-value < 2.2 × 10^-16^), suggesting a substantial enrichment in true FIs relative to an interaction set derived from raw features alone.

### Merging the NBC with pathway data to create an extended FI network

To construct an extended FI network with high protein and gene coverage, we merged FIs predicted from our trained NBC with annotated FIs extracted from five pathway databases. The five pathway databases used were Reactome [23], Panther [60], CellMap [61], NCI Pathway Interaction Database [62], and KEGG [63] (Table 2).

**Table 2:** Pathway data sources in the functional interaction network

Data source	Proteins	SwissProt proteins (coverage)	Interactions	Reference
Reactome	4,490	3,863 (19%)	74,869	[23]
Panther	1,912	1,355 (7%)	33,425	[60]
CellMap	567	567 (3%)	1,195	[61]
NCI Nature	1,492	1,486 (7%)	10,845	[62]
NCI BioCarta	1,137	1,136 (6%)	6,695	[62]
KEGG	2,497	2,261 (11%)	13,934	[63]
TRED	1,167	1,166 (6%)	3,030	[64]

SwissProt coverages were calculated as described in Table 1. NCI Pathway Interaction Database has two divisions: batch-imported pathways from BioCarta [83] and pathways hand-curated by NCI Nature pathway curators. We represent these two divisions separately. TRED is a transcription factor/target database. We have imported the human curated part of transcription factor-target interactions from this database for our network.

To further increase the coverage of our network, we imported interactions between human transcription factors and their targets from the TRED database [64]. TRED has two parts: one contains highly reliable, human curated data from published literature and the other is uncurated and comprises predictions based on several computational algorithms. For our purposes, we used the human curated part only to ensure the reliability of our FI network, and treat these interactions as a part of the pathway FIs in this report.

The extended FI network contains 10,956 proteins (9,393 SwissProt accession numbers, splice isoforms not counted) and 209,988 FIs (Table 3). It covers 46% of SwissProt proteins.

**Table 3:** Protein identifiers and functional interactions in the extended FI network

Source type	Proteins	SwissProt proteins (coverage)	Interaction
Pathways	6,316	5,496 (27%)	98,590
Predicted	8,345	7,546 (37%)	111,398
Total	10,956	9,393 (46%)	209,988

FIs listed in the pathways row include transcription factor-target interactions imported from the TRED database.

The average connection degree (that is, the number of interacting partners per protein) of the extended network is 38, and the maximum degree is 593 for protein P32121 (ARRB2, Beta-arrestin-2). Most proteins in this network are interconnected: 10,645 proteins are interconnected in the largest connected graph component. The remaining 311 proteins reside in 124 connected graph components of size 7 or smaller.

The FI network shows scale-free properties (data not shown) as do other biological networks [65-68]. GO slim annotation enrichment analysis results (not shown) show that our network is enriched in proteins involved in signal transduction, cell cycle and the central dogma. This reflects the ascertainment bias of using Reactome as the training set, as these pathways reflect high priorities for Reactome curation.

### Assessing the utility of functional interactions in the network

GBM is the most common type of brain tumor in humans and also has the highest fatality rate. Recently, two data sets from two independent high throughput screens for somatic mutations involved in GBM have been released [12,14]. In this section, we demonstrate that the interactions from our network can be used to automatically extend a hand-curated GBM pathway developed to support the analysis of one of these data sets [14]; the extended GBM pathway captures more observed somatic mutation events and can be used to generate testable biological hypotheses.

In preparation for analysis of The Cancer Genome Atlas (TCGA) somatic mutation data set [14], a team of bioinformaticians, molecular biologists and clinical oncologists based at Memorial Sloan Kettering Cancer Center and Dana-Farber Cancer Institute developed a human-curated map of the molecular pathways involved in GBM (Figures S7 and S8 in [14]; the original Cytoscape file can be downloaded from [69]). Our network captures the majority of proteins and interactions in this map: 96% of proteins (70 of 73) and 69% of interactions (129 of 187).

The TCGA GBM screen captured 341 mutated genes, including both point mutations and copy number variations (CNVs). Of these genes, 38 (11%) are part of the original hand-curated GBM pathway, and 237 (70%) are in the FI network. Of these genes in the FI network, 36 are in the original GBM pathway (15%), and in addition, 108 directly interact with at least one of the curated GBM pathway genes, for a total of 42% of the somatic mutations. This degree of interaction between somatically mutated genes with the GBM pathway is far greater than would be expected by chance (*P*-value = 1.3 × 10^-23 ^by the hypergeometric test), suggesting that the FI network provides an effective way to enrich the hand-curated GBM pathway for additional genes involved in the disease.

**Figure 3:**
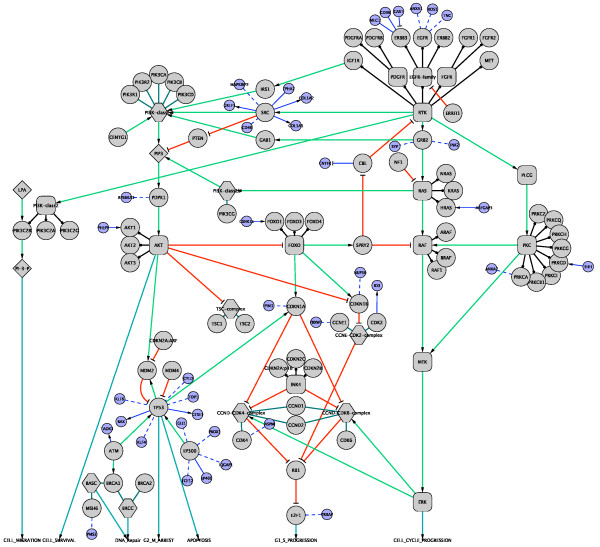
**Overlay of predicted functional interactions onto a human curated GBM pathway from the TCGA data set**. Many genes can interact with multiple pathway genes. In this diagram, only genes interacting with one pathway gene are shown to minimize diagram clutter. Newly added genes are colored in light blue, while original genes are colored in grey. Newly added FIs are in blue, while original interactions are in other colors. FIs extracted from pathways are shown as solid lines (for example, PHLPP-AKT1), while those predicted based on NBC are shown as dashed lines (for example, KLF6-TP53). Extracted FIs involved in activation, expression regulation, or catalysis are shown with an arrowhead on the end of the line, while FIs involved in inhibition are shown with a 'T' bar. The original GBM pathway map in the Cytoscape format was downloaded from [69].

We then added these potential proteins and interactions to the GBM pathway map to extend it. In order to do so, we chose proteins that were found to have one or more somatic mutations in the GBM screen, and had direct interactions with one or more of the proteins in the hand-curated GBM pathway. In this way we were able to extend the hand-curated pathway from 73 proteins and 187 interactions to 181 proteins and 768 interactions. A total of 581 FIs were added between pathway components and new mutated protein interactions (an increase of 148% for proteins and 311% for FIs). Figure 3 shows the original hand-curated map after extending it with predicted and curated FIs from the FI network involving mutated genes. Interactions derived from curated pathways are represented as solid lines (with arrows for FIs involved in catalysis and activation, and with a 'T' bar for those involved in inhibition), while those predicted from the NBC are shown as dotted lines. Many mutated proteins interact with more than one pathway component. For the purposes of readability, Figure 3 shows only proteins that interact with one pathway component. A larger diagram showing the fully extended map is available in Figure S2 in Additional file 1.

A total of 23 of the FIs added to the GBM pathway in Figure 3 were predicted by the NBC. To validate the accuracy of these predicted FIs, we searched the published literature for evidence supporting that two genes in the predicted FIs are indeed functionally related. Table 4 lists the literature references that support these interactions. Out of 23 FIs, a total of 18 (78%) are supported by literature evidence for a functionally significant event. One FI (ROS1-EGFR) has no literature evidence supporting it, and the remaining four are confirmed physical interactions but have no evidence of functional significance. These results suggest that the predicted FIs are sufficiently reliable to be safely integrated into known pathways for systematic analysis.

**Table 4:** Literature references for predicted FIs added to human curated GBM pathway from the TCGA GBM data set

Pathway gene	FI partner	Reference	Comment
*CCNE1*	FBXW7	[99]	Turnover of CCNE1 protein is dependent on FBXW7 protein
*CDK4*	ASPM	[100]	Physical interaction: functional relationship is not clear
*CDKN1A*	PIM1	[101]	Pim-1 kinase dependent phosphorylation of p21Cip1/WAF1 (CDKN1A) influences subcellular localization of p21
*CDKN1B*	NUP50	[70]	NUP50 protein is required for degradation of CDKN1B protein, which is important in cell cycle regulation
*E2F1*	TRRAP	[102]	TRRAP is required as a cofactor for E2F transcriptional activation
*EGFR*	ANXA1	[103]	ANXA1 protein and other annexins are involved in degradation of EGFR protein
*EGFR*	ROS1		This may be a false positive example
*EGFR*	TNC	[71]	TNC protein is a ligand for EGFR
*EP300*	GLI1	[104]	GLI1 is involved in a GLU1-p53 inhibitory loop
*EP300*	IQGAP1	[105]	Physical interaction: functional relationship is not clear
*EP300*	PROX1	[106]	Physical interaction: functional relationship is not clear
*EP300*	TCF12	[107]	Form a functional complex in neurons
*GRB2*	SYP	[108]	SYP involvement in the RAS pathway has been reported some time ago
*GRB2*	TNK2	[109]	TNK2 protein is a target of GRB2 protein
*MSH6*	PMS2	[110]	*PMS2 *has been treated as a DNA repair gene
*PDPK1*	RPS6KA3	[111]	Phosphoserine-mediated recruitment of PDPK1 to RPS6KA3 leads to coordinated phosphorylation and activation of PDPK1 and RPS6KA3
*PRKCA*	ANXA7	[112]	Calcium-dependent membrane fusion driven by annexin 7 can be potentiated by protein kinase C and guanosine triphosphate
*SRC*	CD46	[113]	CD46 is a substrate of SRC
*SRC*	MAPK8IP2	[114]	Though no direct evidence shows a functional relationship between these two genes, it is shown that an isoform of JIP (MAPK8IP2), JIP1, is regulated by Src family kinases
*TP53*	CYLD		CYLD is a deubiquitinating enzyme. Several deubiquitinating enzyme have been shown to be involved in the p53 pathway; however, no evidence has been provided for CYLD in the p53 pathway
*TP53*	KLF4	[115]	KLF4 is a direct suppressor of expression of TP53
*TP53*	KLF6	[116]	Physical interaction: TP53 may enhance the function of KLF6
*TP53*	TOP1	[117]	Activity of TOP1 may be modulated by P53

A detailed examination of the extended GBM pathway can lead to hypotheses that connect the observed sequence alteration in the TCGA data set to known biological pathways. For example, NUP50 is required for degradation of CDKN1B protein [70]. Copy number deletion in *NUP50*, which occurs in three TCGA GBM samples, may inhibit the degradation of CDKN1B and impact the cell cycle process. For another example, tenascin-C (TNC) protein is a ligand for epidermal growth factor receptor (EGFR) [71]. Three re-sequenced GBM samples have found TNC mutations, which may disturb the RTK/RAS signaling pathway via its interaction with EGFR.

It needs to be pointed out that the directionality of the interaction should be taken into account when using the FI network to frame hypotheses. For example, two of the pathway FIs around TP53, BAX-TP53 and GTSE1-TP53 were originally extracted from the KEGG human p53 signaling pathway [72]. The *BAX *and *GTSE1 *genes are transcriptionally upregulated by TP53 protein. Though it is not annotated in the original KEGG database, there is evidence showing that GTSE1 protein can regulate TP53 protein's activity and localization [73]. However, there is no evidence to suggest that the P53 pathway is affected by BAX protein, a protein involved in apoptosis [74]. Hence, mutations in BAX in a particular tumor do not support an etiology involving P53 signaling, but instead might point to events downstream of P53. The same caveat applies to predicted FIs as well.

### Clustering of GBM sequence-altered genes in the extended FI network

The previous section described how the FI network can be used to enhance and extract novel hypotheses from a previously created hand-curated disease pathway. In this section, we illustrate how studies of distributions of altered genes in the GBM samples in the FI network can assist in genome-wide functional analysis when a preexisting disease pathway is unavailable.

Both the TCGA [14] and Parsons *et al. *[12] GBM studies identified recurrent patterns of somatic gene mutations involving multiple classical signaling pathways using a manual process of inspection and correlation to the literature and a variety of pathway databases. Here, we use network community analysis to automatically identify network modules that contain genes and their products that are involved in common processes.

**Figure 4:**
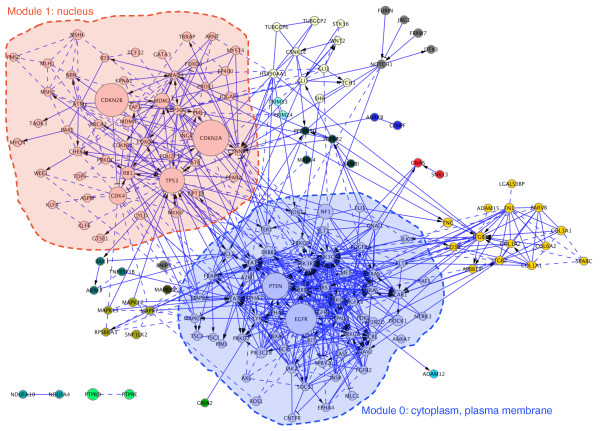
**Edge-betweenness network clustering results for the altered genes from the TCGA data set**. Gene nodes in different clusters are displayed in different colors. GO cellular component annotation for clusters 0 and 1 are labeled in the diagram to show the major cellular localizations for genes in these two clusters. The node size is proportional to the number of samples bearing displayed altered genes.

The edge-betweenness algorithm [75] has been used to find network modules in protein interaction networks [76-78]. We applied this algorithm to search for FI network modules for sequence-altered genes identified in the two GBM data sets. Starting with the TCGA data set, we collected 341 mutated and CNV genes from 91 GBM samples that have been re-sequenced in that study. A total of 237 of these genes (70%) were in the FI network. Of these, 168 have mutual FIs and are interconnected. We built a subnetwork around these 168 genes, applied the edge-betweenness network clustering to it, and obtained 17 network modules, 6 of which were greater than size 4 (Figure 4).

The sizes of the first two modules (modules 0 and 1) are 63 and 50, respectively. The distribution study showed that 76 out of 91 GBM samples have altered genes in both module 0 and module 1 (84%, *P*-value < 1.0 × 10^-4 ^from permutation test). As a cross-validation test, we projected 22 samples from the discovery screen in the Parsons data set, which provided both somatic mutation and CNV data, onto these network modules. The result showed that 68% (15 out of 22) have altered genes in both module 0 and module 1 from the TCGA data set (*P*-value < 1.0 × 10^-4^). We also did a reciprocal test by applying the edge-betweenness clustering algorithm to a subnetwork composed by altered genes from the Parsons data set, and checking sample distributions from both GBM data sets in the network modules. The results are similar to our results in the TCGA data set: 77% (*P*-value = 0.0002) of GBM samples in the Parsons data set, and 71% (*P*-value < 1.0 × 10^-4^) in the TCGA data set have altered genes in two corresponding modules (Figure S3 in Additional file 1).

To see what biological features these two modules may connote, we annotated these two modules using pathways and GO terms. GO cellular annotation enrichment assay indicated that module 0 mainly corresponds to proteins present in the cytoplasm and plasma membrane, while module 1 mainly involves gene products present in the nucleus. Many pathways can be assigned to these two modules, but it is clear that module 0 is mainly related to signaling transduction pathways while module 1 is related to the cell cycle, DNA repair and pathways involved in chromosome maintenance (Table S2 in Additional file 1). The fact that most of the GBM samples have altered genes in both modules implies that these two major modules are acting cooperatively in establishing and/or maintaining the GBM phenotype, and suggests that the development of GBM cancers involve malfunctions in both signaling transduction and cell-cycle regulation.

Our FI network is composed of a combination of curated FIs and predicted FIs. To determine whether the distribution of altered genes is robust, we checked the above results against FI network modules composed of FIs derived from curated FIs only. The results are similar to those obtained using the integrated FI network except that network modules 0 and 1 are smaller than the modules built with both predicted and pathway FIs (results not shown). Figure 4 shows that many mutated genes are brought into modules 0 and 1 based on predicted FIs only, which are shown with dashed lines.

**Figure 5:**
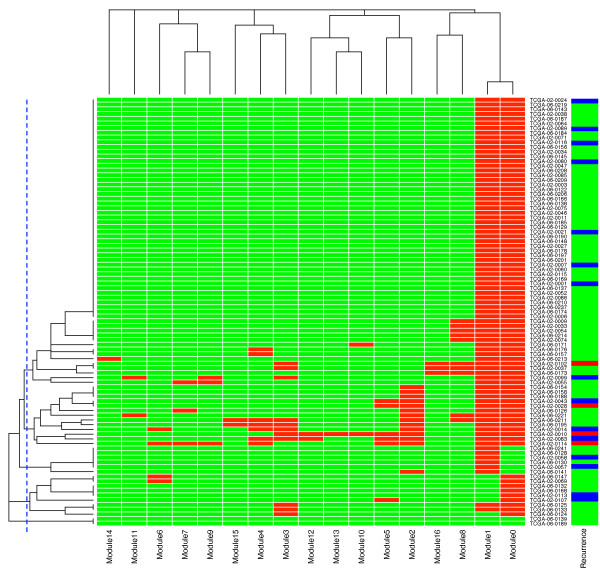
**Hierarchical clustering of GBM samples in the TCGA data set based on altered gene occurrences in the network modules identified by the edge-betweenness algorithm**. The rows are samples, while the columns are 17 network modules. In the central heat map, red rectangles represent samples having altered genes in modules, while green rectangles represent samples having no altered genes in modules. The vertical blue dashed line shows the cutoff value we used to select sample clusters from the hierarchical clustering. The right-most column lists sample types: green for primary GBM samples ('No' in Table S1B in [14]), blue for recurrent ones ('Rec' in Table S1B in [14]), and red for secondary ones ('Sec' in Table S1B in [14]).

To further explore the distribution of mutations among the network modules, we performed a hierarchical clustering of the TCGA GBM samples based on the occurrence of altered genes in the modules (Figure 5). From this clustering, we obtain five sample clusters of size 61, 13, 6, 9, and 2, respectively. Three types of samples were used in the original TCGA screening (rightmost column of Figure 5): recurrent samples (15, blue), secondary samples (4, red), and primary samples (72, green). Sample cluster 0, which has a signature of mutations in both network modules 0 and 1, is enriched in primary tumor samples (*P*-value = 0.055 from Fisher test). In contrast, sample cluster 1, which has additional mutations involving network modules 8, 3, 9, 7 and others, is enriched in samples from tumor recurrences and metastases (*P*-value = 0.026). Indeed, all but one of the four metastatic samples can be found in sample cluster 1 (*P*-value = 0.0086).

In the original TCGA paper [14], seven of the recurrent or metastatic samples were labeled as 'hyper-mutated' because of their much higher rate of somatic mutation. We found that except for one sample (TCGA-02-0099) located in sample cluster 0, all of the other six samples are in cluster 1 (*P*-value = 1.7 × 10^-5^). These results illustrate how the mutated network modules can be used to differentiate cancer samples.

### Defining a GBM core cancer network

**Figure 6:**
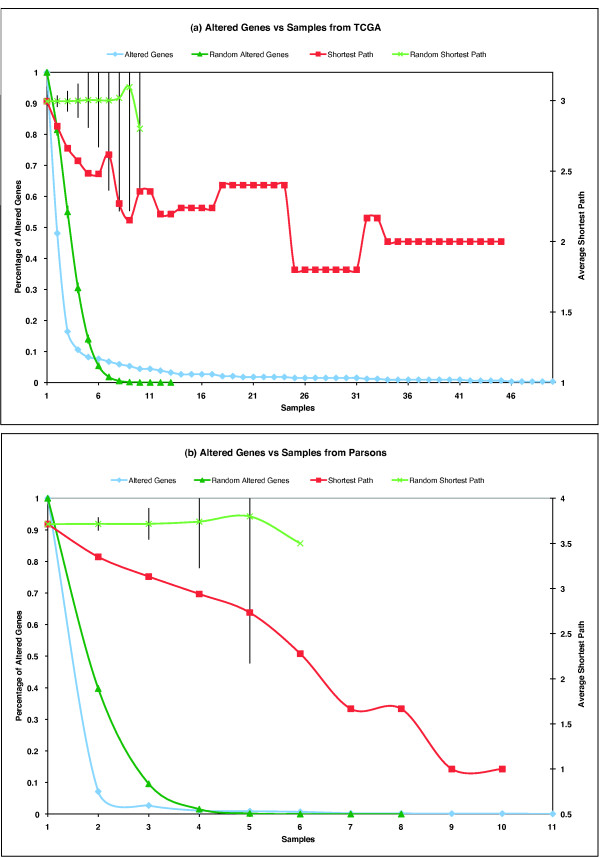
**Plots of altered genes versus samples**. The horizontal axis is the sample numbers, and the left vertical axis is the percentage of altered genes occurring in samples related to total altered genes. The right vertical axis is the average shortest path among altered genes occurring in samples. **(a) **The TCGA data set. **(b) **The Parsons data set.

It is expected that multiple false positive ('passenger') genes exist in the set of sequence-altered genes identified from the GBM samples. It is also expected that true positive ('driver') GBM-related genes should occur more often in GBM samples than by chance. We plotted the percentage of altered genes versus samples for both GBM data sets (Figure 6), and compared this distribution against what would be expected by random assignment of genes to samples. There are two phases in the distribution of altered genes across samples. In the first phase, involving gene alterations occurring between two to five samples, there is sharing of fewer altered genes than would be expected by chance. In the second phase, involving genes altered independently in six or more samples, there are more altered genes shared among the samples than would be expected by chance. This result can be explained if there exist a minimum number of driver genes that must be mutated in order to produce GBM, and that this 'GBM core' tends to be recurrently mutated in independent samples. Figure 6 also shows that the average shortest path among shared genes from GBM samples decreases versus sample numbers in contrast to random samples, which implies that GBM candidate genes tend to be closer in the FI network than by chance (see below).

**Figure 7:**
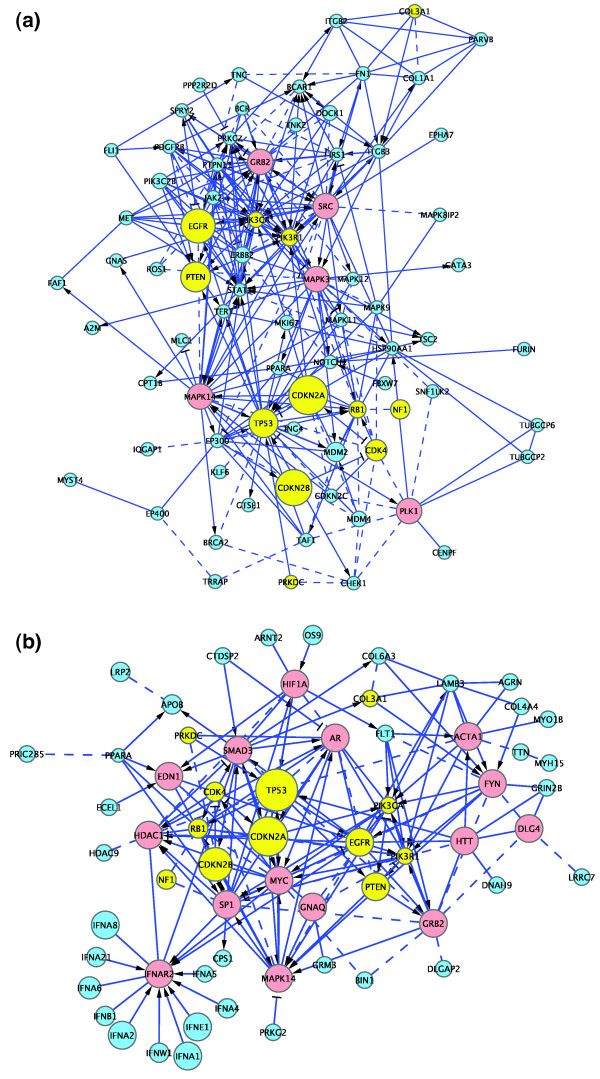
**Subnetworks for GBM clusters**. **(a) **The TCGA cluster. **(b) **The Parsons cluster. Shared GBM candidate genes are shown in yellow, non-shared candidate genes in aqua, and linker genes used to connect cancer genes in red. The node size is proportional to the number of samples bearing displayed altered genes. Other colors and symbols are as in Figure 2.

To visualize sequence-altered genes and further define the core set of genes in the GBM samples, we collected genes altered in at least two samples to reduce the number of false positive GBM candidate genes, performed hierarchical clustering among them to identify a set of highly interconnected candidates, and then selected and built subnetworks containing >70% of altered genes (Figure 7a, b) by adding the minimum number of linker genes to form a fully connected subnetwork.

In the TCGA data set, 164 altered genes occurred in two or more GBM samples, 98 (60%, *P*-value = 3.2 × 10^-7^) of which were in the FI network. Of these, 71 are in the GBM subnetwork (72%, *P*-value < 0.001 from permutation test). An average shortest distance calculation (Table 5) shows that genes in this cluster are linked together much more tightly than would be expected by chance: 2.29 for subnetwork genes versus 3.83 for a similarly sized random set of genes treated in the same way as the cancer subnetwork. In the Parsons data set, 111 genes occur in two or more GBM samples, 65 (59%, *P*-value = 8.4 × 10^-5^) of which are in the FI network. Of these, 46 are in the GBM cancer cluster (71%, *P*-value < 0.001 from permutation test). Similar to the TCGA data set, the average shortest path among these genes is shorter than by chance (2.76 versus 3.82, *P*-value < 0.001).

**Table 5:** Clustering of GBM-related altered genes occurring in two or more samples in the FI network

Data set	Cancer genes	Genes in FI network (%)	Genes in cluster	Percentage of genes in cluster (*P*-value via random permutation)
TCGA	164	98 (60%)	71	72% (<0.001)
Parsons	111	65 (59%)	46	71% (<0.001)

The numbers in the 'Cancer genes' column are for altered genes in two or more samples from the TCGA and Parsons data sets. Genes were collected using both somatic mutations and CNVs.

In the average shortest path calculation, a potentially confounding factor in the TCGA data set is that 601 genes pre-selected for sequencing may be more tightly interconnected than average. Indeed this is the case. When we performed the permutation test using these 601 pre-selected genes, we obtained an average shortest path of 2.40, which is shorter than the genome-wide average, but still longer than the length of 2.29 calculated for the subnetwork formed by recurrently mutated genes (*P*-value = 0.023; connection degrees have been considered in permutation test (see below)). This consideration does not apply to the Parsons set, which used an unbiased resequencing approach.

In summary, results from both GBM data sets indicate that more than 70% of the recurrently mutated genes are more tightly interconnected than expected by chance, and occupy a small corner of the large FI network space.

We found that the average connection degrees in the GBM clusters are higher than the average connection degree in the whole FI network (40 based on the biggest connected graph component using gene names): 87 for the TCGA cluster (*P*-value = 1.3 × 10^-5 ^from *t*-test), and 60 for the Parsons cluster (*P*-value = 0.13). The result that the average shortest path among altered genes in cancer clusters is shorter than by chance may be an ascertainment bias due to the higher connection degrees in the cancer clusters resulting from the intensive study of signal transduction pathways, to which most GBM candidate genes belong. To determine whether the differences in average shortest paths between the cancer clusters and randomly selected genes are due entirely to the difference in degree, we performed an additional permutation test in which the genes picked were stratified by degree in order to match the distribution of the cancer gene sets (Table 6, Degree-based permutation column). This correction reduced, but did not eliminate, the differences in average shortest path between the cancer gene sets and randomly selected genes, and the differences remained statistically significant: both *P*-values < 0.001.

**Table 6:** Average shortest distance for GBM clusters

Data set	Cancer genes	Random permutation (*P*-value)	Degree-based permutation (*P*-value)
TCGA	2.29	3.83 (< 0.001)	2.86 (<0.001)
Parsons	2.76	3.82 (< 0.001)	3.32 (<0.001)

The values in the 'Cancer genes' column are from the cancer clusters, while those in the two permutation columns are from permutation tests. Random permutation is done via randomly picking up the same number of genes from the biggest connected graph component as for the cancer genes, and degree-based permutation is done by picking up genes from different bins based on the degree distributions of cancer genes. The gene bins are dynamically generated based on the sorted list of degrees from the cancer genes.

The reason why the average shortest paths for the TCGA data set are smaller than the those calculated for the Parsons set for both the cancer cluster and degree-based permutation results is that re-sequenced genes in the TCGA data set number 601 in total, which are pre-selected and believed to be more cancer-related, while the Parsons paper resequenced 20,661 protein coding genes.

**Figure 8:**
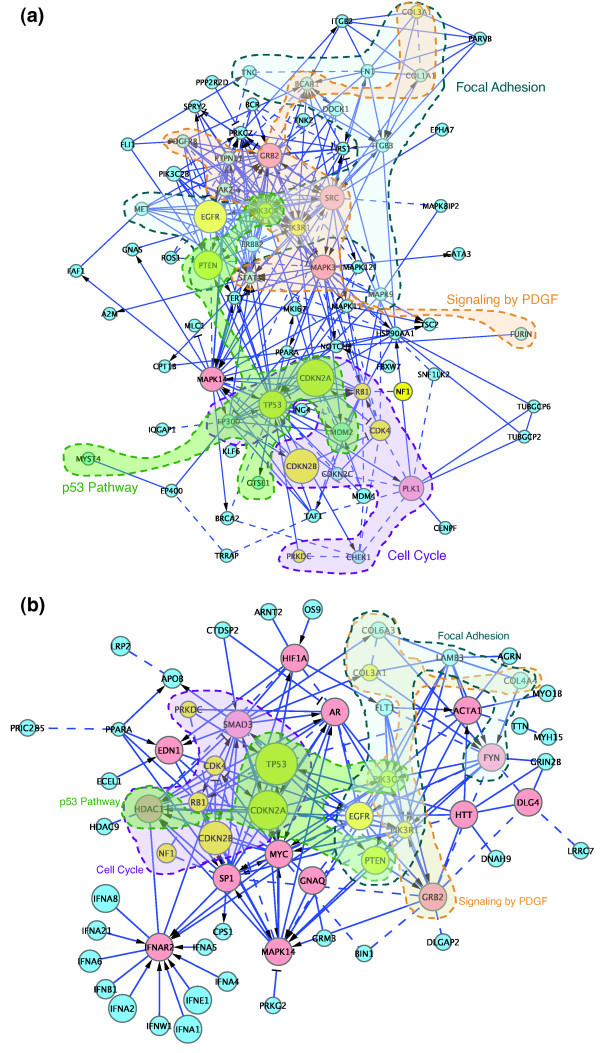
**Subnetworks with pathways annotated for GBM clusters**. Many pathways are hit by GBM candidate genes. Only four of them are labeled for two GBM clusters in this diagram to simplify the diagram. Colors and symbols are as in Figure 6.

Looking at the two GBM cancer subnetworks in more detail, each subnetwork consists of GBM candidate genes ('cancer genes') plus the minimum number of interacting genes necessary to interconnect them ('linker genes', shown in red in Figures 7 and 8). The TCGA subnetwork contains 77 genes, 5 of them linkers, while the Parsons subnetwork contains 62 genes, 14 of them linkers. Since many of our FIs were extracted from human curated pathways, it is easy to superimpose pathways back to these subnetworks to see what pathways are involved in these cancer genes. Many pathways are statistically significantly hit by these genes. Figure 8 shows four pathways: focal adhesion, signaling by platelet-derived growth factor (PDGF), p53, and cell cycle (false discovery rate <8.0 × 10^-4^). As illustrated in Figure 8a, b, there are extensive overlaps among, and cross-talk between, these pathways.

It is surprising that the two GBM studies identified a relatively small number of GBM candidate genes in common: just 15 out of a total of 260 unique genes are shared (6%) for all GBM candidate genes (13 out of a total of 150 unique genes (9%) for altered genes in the FI network). Intriguingly, 12 out of 13 shared genes in the FI network are present in the two cancer subnetworks we built independently for the two data sets (Figures 7 and 8, shared genes are in yellow; *P*-value = 0.0014 based on permutation test), suggesting that the GBM cancer clusters capture the common candidate genes. Interestingly, with the exception of *COL3A1*, all the shared genes directly interact with each other, suggesting that they form the core of a GBM pathway, and that non-shared cancer genes are extensions of the core network.

To further narrow down the list of candidate genes to those that are likely to be drivers, we used the results shown in Figure 6 to investigate candidate genes altered in eight or more GBM samples in the TCGA data set, and five or more samples in the Parsons data set. In the TCGA data set, a total of 20 genes are altered in 8 or more GBM samples. Of these 20 genes, 13 are in our FI network, and 10 are displayed in Figure 7a: *CDK2A*, *CDK2B*, *CDK4*, *EGFR*, *MDM2*, *NF1*, *PTEN*, *RB1*, *PIK3R1*, and *TP53*. In the Parsons data set, 14 genes occur in 5 or more samples, 10 are in the FI network, and 9 are displayed in Figure 7b: *CDKN2A*, *CDKN2B*, *EGFR*, *IFNA1*, *IFNA2*, *IFNA8*, *IFNE1*, *PTEN*, and *TP53*. Out of these genes, five are shared between these two data sets: *CDKN2A*, *CDKN2B*, *EGFR*, *PTEN*, and *TP53 *(*P*-value = 5.8 × 10^-13^). The fact that these genes are altered in multiple samples and shared in two studies further indicates the existence of a GBM core network.

### Application of the FI network to other cancer types

In this section, we test whether the network patterns found in the GBM samples can also be found in other cancer types. We applied our FI network to the genome-wide data sets for breast [10], colorectal [10] and pancreatic cancers [11], and found similar patterns of clustering of mutations into network modules.

In the breast and colorectal data sets, 18,191 genes based on 20,857 transcripts were re-sequenced for 11 breast and 11 colorectal tumors during the discovery phase. Mutated genes found in the discovery phase are further validated with 24 additional samples of the same tumor type. For network module analysis, we collected mutated genes from the 11 discovery phase samples from both breast and colorectal cancers, built interaction subnetworks based on the FI network, clustered the subnetworks using edge-betweenness, and compared the resulting network modules to the distribution of patient samples.

For breast cancer samples, 1,023 genes were mutated in 11 tumor samples. Of these, 524 genes are in our FI network (51%). The edge-betweenness algorithm generated 33 clusters, of which 12 were of size 5 or greater. The sizes of the first three modules are 55 (module 0), 49 (module 1), and 26 (module 2) (Figure S4 in Additional file 1). The sample distribution analysis showed that all 11 breast samples had mutated genes in modules 0 and 1, and 9 out 11 breast tumor samples had mutated genes in modules 0, 1, and 2. A GO cellular component annotation enrichment analysis showed that genes in module 0 are mainly from the extracellular region, cytoplasm and the plasma membrane, while genes in module 1 are found mainly in the nucleoplasm, nucleus and nucleolus (Table S3 in Additional file 1). Pathway annotation shows that genes in module 0 are involved in integrin and focal adhesion pathways, while genes in modules 1 and 2 are involved in transcription, cell cycle and DNA repair (Table S3 in Additional file 1).

For colorectal cancers, 766 genes were mutated in 11 tumor samples. Of these, 410 genes are in our FI network (54%). We detected 31 modules from the edge-betweenness algorithm, 8 of which were of size 5 or greater. Nine out of 11 colorectal cancer samples had mutated genes involving three network modules, modules 0, 1 and 4 (Figure S5 in Additional file 1). A GO cellular component enrichment analysis (Table S4 in Additional file 1) demonstrated that module 0 is enriched for plasma membrane and cytosol, while module 1 is enriched in GO terms describing the extracellular region, and module 4 is enriched in terms for the nucleoplasm and nucleus. Compared with breast cancer, module 0 in breast cancer has been split into two modules in colorectal carcinoma. Pathway annotation showed that genes in module 0 are highly enriched in components of the endothelin and Nerve Growth Factor (NGF) pathways, in contrast to breast cancer and GBM, which show no such enrichment. Genes in module 1 are involved in focal adhesion and integrin pathways, while genes in module 4 are involved in DNA repair (Table S4 in Additional file 1), all of which are also enriched in breast cancer and GBM.

As with the two GBM data sets, we performed a hierarchical clustering analysis on genes mutated in at least two or more samples. In this analysis, we used samples from both the discovery and validation phases to increase the sample numbers. Table S5 in Additional file 1 lists the numbers of genes mutated in two or more samples, mutated genes in the whole FI network, and mutated genes in the clusters from hierarchical clustering; these numbers indicate that most of the mutated genes are clustered together in the FI network, as was previously observed with GBM candidate genes. Also similar to the results from the GBM analysis, the majority of cancer candidate genes in both breast and colorectal cancers are closer than would be expected by chance (Table S6 in Additional file 1). Figures S6 and S7 in Additional file 1 show these clusters with their pathway annotations. As with GBM, many cancer-related pathways are significantly enriched in these clusters.

We performed a similar network module and hierarchical clustering analysis on a pancreatic data set [11]. Our results show that similar network patterns can be found in pancreatic cancers (Tables S5, S6, and S7, and Figures S8 and S9 in Additional file 1). We noted that 75% (*P*-value = 0.38) of pancreatic samples (18 out of 24 samples) have mutated genes in module 0 (nucleus and cytosol) and module 1 (extracellular region), and 71% (*P*-value = 0.24) of samples (17 samples) have mutated genes in module 0 and module 2 (plasma and integral to membrane) (Figure S8 in Additional file 1).

As in the GBM analysis, we used permutation testing to assess whether more of the breast, colorectal, or pancreatic cancer samples had mutations involving these modules than would be expected by chance. However, due to the small number of samples available and the relatively large number of mutations per specimen (32 to 49 altered genes per specimen in the breast, colorectal and pancreatic sets, versus 22 per specimen in GBM), we failed to demonstrate statistically significant overrepresentation of specimens with mutated genes in these modules.

In conclusion, we found that most samples from breast, colorectal or pancreatic cancers have sequence-altered genes involving multiple modules localized in different cellular localizations, and that most cancer candidate genes in the FI network are closer than would be expected by chance. The results are broadly similar to those seen with GBM, but there are many intriguing differences as well in the identity of the pathways involved.

### Software tools for accessing the functional interactions

**Figure 9:**
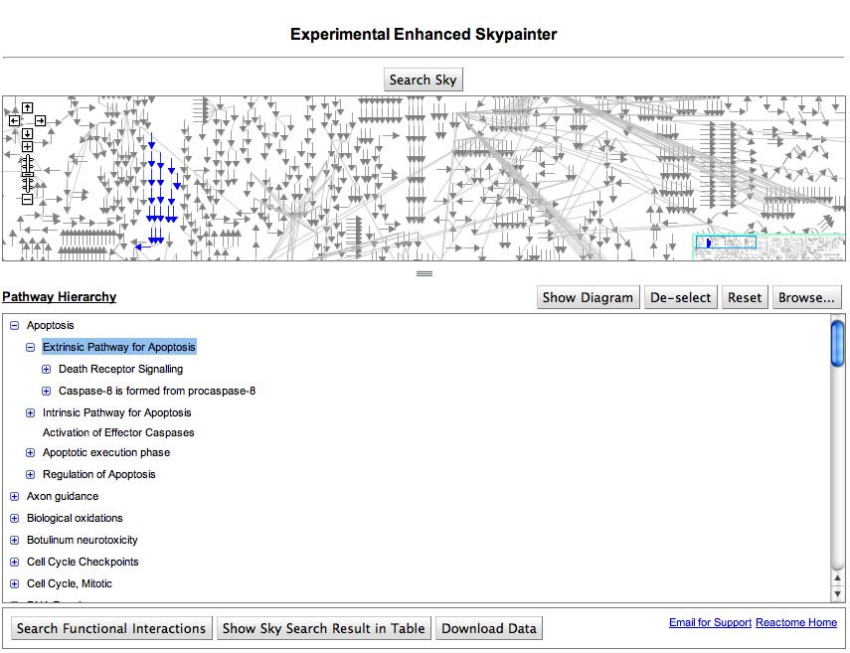
**Front page of the web application for predicted functional interactions**.

To make our FI data sets available to general researchers, we developed a web-based application. This application was built upon the current Reactome SkyPainter using Google Web Toolkit (GWT) and is available at [79]. Figure 9 is a screenshot of the application when first launched. At the top of the application is a Google-map style view of all reactions manually curated for Reactome; at the bottom is a hierarchical tree of all pathways, organized by biological process.

**Figure 10:**
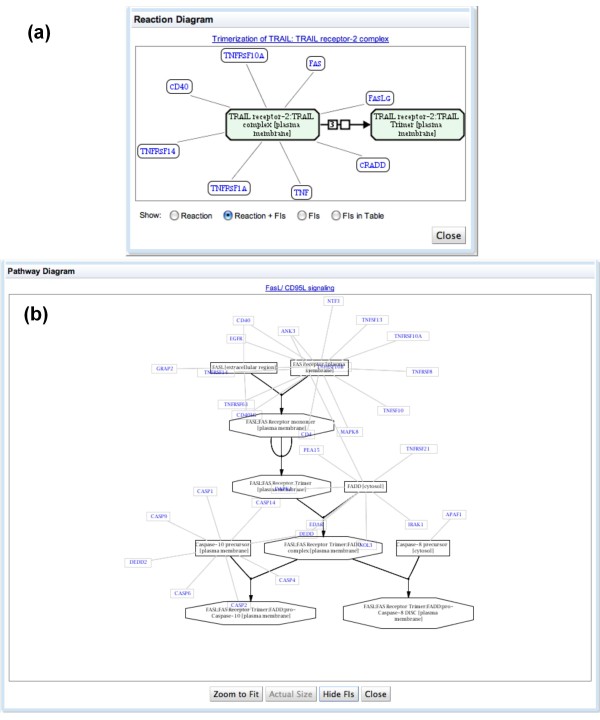
**Views of predicted functional interactions**. **(a) **FIs in a reaction diagram. **(b) **FIs in a pathway diagram.

To find FIs, users can upload lists of UniProt accession numbers or gene names for proteins or genes of interest. The application will then highlight pathways involving FIs with the proteins or genes of interest in both the reaction map and the pathway hierarchy. When the user selects a reaction that has been 'hit', a dialog appears that provides detailed information on the relationship between the reaction, its components, and FIs involving those components (Figure 10a). The user can also select a hit pathway in the hierarchical tree to bring up a pathway diagram to show pathway components and its FIs (Figure 10b).

The entire set of FIs can be downloaded as a MySQL database dump (Additional file 2) or in tab-delimited files (Additional file 3) from this web application. There is no restriction on their use or redistribution beyond citing the source of the information.

## Discussion

Increasingly, human diseases and other traits are being probed by genome-wide screens. For example, several recent papers [10-14] describe genome-wide screening efforts to identify somatic mutations in several cancer types. Placing such lists of genes or proteins into a pathway context can yield information on the relationships among these genes and has the potential to generate hypotheses about the mechanism(s) linking these genes to phenotypes.

Reliable pathway databases are essential for such an analysis, but because of the effort needed to curate pathways is so human-intensive, even the largest pathway database has a SwissProt coverage of under 20% (Table 2). In this report, we describe how we have integrated several large-scale experimental data sets to build and train a machine-learning system that identifies potential 'functional interactions' among pairs of human proteins. We have combined the FIs predicted by this classifier with the curated pathways from Reactome and other pathway databases to create an extended database of FIs that covers nearly 50% of the human proteome. Our literature search for a small set of predicted FIs around a human curated GBM pathway [14] shows that our prediction has an accuracy around 78% (Table 4).

An essential step in building this extended FI network was the construction of a naïve Bayes classifier to predict FIs from a combination of physical PPI data and other noisy sources of information.

NBC is a simple, robust method that is usually the first to be tried in machine learning techniques. NBCs can frequently yield better results than more sophisticated techniques [80]. One requirement for a successful NBC is that the features used in the classifier be independent. In our NBC, we used human physical PPIs, protein-protein interologs from yeast, worm, and fly, two independent gene co-expression data sets, protein domain interactions, GO biological process annotations and PPIs generated from text mining. Human PPIs, protein interactions from other species, and gene-expression data sets were generated experimentally, and meet the requirement for independence. Many of the GO annotations and protein domain interactions are based on sequence similarities among proteins in different species. Hence, there is a potential dependency among these two data types and protein-protein interologs from yeast, worm and fly since they all rely on the same phylogenetic trees. Many human protein interactions in interaction databases we used (IntAct, BioGrid, and HPRD) are human curated from the literature. This literature may be used by the text mining technique to generate PPIs. But we feel both effects are likely to be small.

Many computational methods have been developed to generate biological pathways and networks based on DNA microarray or other similar data sets from scratch with promising results [81]. Most recently, Vaske *et al. *[82] developed a factor-graph-based method to expand known pathways using array data sets generated from perturbation experiments. Here we propose a new approach to expand a known human curated pathway by using FIs, with the example of the human curated GBM pathway. Compared to the computational approach, our method provides direct supporting evidence for expanded FIs from curated pathways (for curated FIs) and high reliability from multiple data sources (for predicted FIs). We are in the process of developing a software tool so human curators can make use of these FIs to expand known pathways for expedited curating.

By applying our FI network to two independent GBM data sets from whole-genome screening projects, we are struck by the finding that most genes with recurrent mutations (>70%; Table 6) co-cluster into a small corner of huge FI space. These clusters are highly enriched in classical signaling pathways as well as the cell cycle, in agreement with pathway analyses performed by the original authors of the studies. We are also able to identify extensive crosstalk among the pathways, which indicates the complexities in tumorigenesis. Furthermore, we show how the FI network can reveal overlaps - and possibly common mechanisms - between the two GBM studies. This suggests a scenario in which the two cancer whole-genome screening projects are sampling from a common core cancer pathway that can be revealed by FI network analysis.

Our result that most cancer candidate genes are clustered together is similar to what was reported by Cui *et al. *[83] based on a much smaller signaling interaction network generated from BioCarta [84] and CellMap [61], a small subset of our imported pathway databases. The reason why most cancer genes cluster closely together is still under investigation. The connection degree contributes to such clustering. However, the degree alone still cannot interpret the clustering based on our degree-based permutation test. We suspect that the major factor that governs the clustering is from FIs among cancer genes. A subset of cancer genes may form a small graph component via these FIs in the huge FI network. Such a small graph component may be used as a core to pull other cancer genes together to form a bigger cluster.

Lin *et al. *[85] investigated network patterns for breast and colorectal cancers using a similar but smaller data set [86], and predicted that over half of the mutated proteins (59 out of 83) in breast cancers participate in an interaction cluster, but only a very small percentage of mutated proteins in colorectal cancers form an interaction cluster, which contains 12 proteins. We used different network analysis approaches based on a larger and more reliable FI network. Our results uncovered network modules that have been mutated in the majority of cancer samples and show that most recurrently mutated genes form a network cluster that is more interconnected than would be expected by chance in both breast and colorectal cancers. The results from multiple cancer types imply that the patterns revealed in our study might be common in all cancer types.

Multiple sources of evidence show that tumorigenesis in human is a multi-step process and that genomes of tumors have sequence alterations at multiple sites [1]. Pathway analysis indicates that many pathways are mutated in cancer samples [2]. A striking finding from our study is that, for all cancer types examined so far, most samples have mutations involving a small number of discrete network modules. One of these modules typically corresponds to cell cycle regulation, DNA repair, and other nucleus-based processes, while another corresponds to signal transduction events in the plasma membrane and cytoplasm. This result suggests that the transformation from normal cells to malignant cells requires functional mutations in both nuclear and cytoplasm/plasma membrane-based pathways. However, our work also suggests that different cancer types have different network modules. A detailed network module based comparison analysis is likely to reveal different specific mechanisms in different cancer types.

A major motivation for this work was the desire to integrate information from multiple pathway databases in order to reduce the fragmentation of knowledge stored in these useful resources. Even with common data models such as BioPAX [87], this is not easy to accomplish due to different focus of interests among the pathway databases, and different standard operating procedures, which allow the same series of biological reactions to be described quite differently from one database to the next. By reducing the pathway databases into a series of pairwise FIs, however, we have been able to merge five of the major pathway databases into a single uniform data model, although much information about the distinct roles of each protein has been lost during the process. Much of our current and future effort will be devoted to developing methods to map the FIs back to their original pathway contexts in order to find causal and directional relationships among the proteins.

## Conclusions

We have built a FI network that covers close to half of human gene products. This functional network, which interconnects with the curated pathways available from Reactome and other human curated pathway databases, forms the foundation for a pathway-based data analysis system for high-throughput data analysis. We have applied this system to the analysis of two genome-wide GBM data sets and data sets from other cancer types and revealed common network patterns in cancer related genes and samples, suggesting that there exists a core network in GBM tumorigenesis.

## Materials and methods

### Importing data from non-Reactome pathway databases

Data from four non-Reactome human-curated pathway databases were imported into the Reactome database (28 March 2009 release). These four databases are: Panther [27], CellMap [61], NCI Pathway Interaction Database [62], and KEGG [26]. To store these imported data into the Reactome database, the original Reactome schema was extended by adding one new class, Interaction, as a subclass to Event, and a new attribute, dataSource, to the top-most class DatabaseObject. The latter is used to track the original data sources of imported instances. The data formats used for importing were: BioPAX [87] for CellMap (released June 2006) and NCI Pathway Interaction Database (released March 2009), SBML [88] with Cell-Designer additions for Panther (version 2.5, August 2008), and KGML for KEGG (released on March 8 2009). After importing, all data from Reactome and the four external databases were maintained in a database, which was also used to store PPI data (see below). We have also imported human transcription factor and target interactions from the TRED database [64]. There are two types of data in the TRED database: human curated data from the published literature and predictions based on computational methods. Only the human data were imported to ensure high quality. Panther uses protein families generated from hidden Markov models for pathway annotations [60]. We only imported human UniProt proteins that could be mapped to pathway components reliably based on evidence codes used in the mapping file.

### Importing protein-protein interaction datasets

Human PPIs were extracted from three PPI databases: BioGrid [29] (release 2.0.50, February 2009), HPRD [31] (released August 2007) and IntAct [33] (released March 2009). Data dumps in PSI-MI version 2.5 format from these three databases were processed by an in-house Java PSI-MI parser, converted into the extended Reactome data format, and stored in the extended Reactome database. An odds ratio analysis was used to check the correlation between a PPI or other pairwise data set and FIs extracted from Reactome pathways. The control groups were generated from random pairs by using proteins from the Reactome FIs. The reported odds ratio values in the results section were based on ten permutations.

PPIs in *S. cerevisiae*, *C. elegans *and *D. melanogaster *PPI data sets were downloaded from IntAct (released March 2009). Ensembl Compara [48] was used to map non-human proteins onto putative human orthologs.

### Other data sets for naïve Bayes classifier

The protein domain-domain interaction data set was downloaded from pFam [52] (release 23.0, July 2008).

Two microarray co-expression data sets were used in the NBC: one downloaded from [89], which was compiled from 60 data sets that contained a total of about 4,000 microarrays [49], and another generated by Prieto *et al. *[50].

Protein GO annotations were downloaded from [90] (gene_association.goa_human, released March 2009). A PPI data set generated from a text-mining technique was kindly provided by Rzhetsky *et al. *[53].

### Training of naïve Bayes classifier

We used a NBC to score protein-protein pairwise relationships. These pairwise relationships were extracted from nine data sources described above and used as features to train the NBC. We used a closed-world hypothesis [91] to assign values to a protein pair: if there was a pairwise relationship between the two proteins in the data set, we used true, otherwise false.

To integrate protein-protein pairwise relationships into the pathway context, we extracted pairwise relationships from reactions and complexes annotated in pathways by defining a FI as an interaction in which two proteins are involved in the same reaction as input, catalyst, activator and/or inhibitor, or as components in a complex. To train the NBC, we extracted a positive data set from Reactome using this definition, and filtered out pairs that do not have any feature. We generated a negative training set using random pairs from proteins in the positive data set.

### Clustering of cancer genes in the functional interaction network

Two human GBM data sets [12,14] were used in our cancer data analysis. For sequence-altered genes in GBM, we chose mutated genes and CNV genes for each sample. Many genes exist in CNV chromosome fragments. For our study, we chose those genes that have been labeled 'Genes with gene expression correlated with copy number' in the TCGA data set (Table S3 in [14]) or 'Candidate target' and 'Other genes' in the Parsons data set (Tables S5 and S6 in [12]). Note that CNV genes in the TCGA data set have been pre-filtered based on gene expression data sets, while CNV genes in the Parsons have not since only SAGE expression data are available, which were not used for filtering because of their lower sensitivity for under-regulated genes. To get CNV genes for each sample in the TCGA data set, we used a file, TCGA-GBM-RAE-genemap-n216-20080510-dscrt.txt, downloaded from [92], which lists CNVs for individual samples [93]. For edge-betweenness network clustering [75], we lumped all sequence-altered genes from samples together, searched FIs among these genes, and constructed a subnetwork based on these interactions. A Java graph library, Jung2 [94], was used for edge-betweenness network clustering. Hierarchically clustering of TCGA GBM samples was done using the hclust method in R [95] based on the complete linkage method with the binary distance between binary vectors generated for each GBM sample according to occurrence of altered genes in network modules identified by the edge-betweenness algorithm. The heat map from hclust was drawn using the R package heatplus [96].

To search for GBM cancer clusters, we collected sequence-altered genes occurring in two or more samples (GBM candidate genes), calculated pairwise shortest paths among genes in the FI network, hierarchically clustered them based on the average linkage method, and then selected a cluster containing more than 70% altered genes. To estimate *P*-value for GBM cancer clusters, we did a 1,000-fold permutation test by randomly choosing the same numbers of genes from the biggest connected graph component as the GBM candidate genes to subject to the same hierarchical procedures for the candidate genes.

To construct a subnetwork spanning a set of genes, we implemented a search algorithm guided by the hierarchical clustering results based on shortest path between two clusters in order to keep the number of linking genes to a minimum. To calculate the *P*-value for the average shortest distance for cancer clusters, we performed a 1,000-fold permutation test by randomly selecting the same numbers of genes from the biggest connected graph component. To check if the connection degree is a confounder to clustering of cancer genes, we repeated this analysis after dynamically generating gene bins based on the sorted list of degrees in the cancer genes, and then randomly choosing genes from these bins in the same distribution as the cancer genes.

For other cancer types, we used Table S3 from [10] for somatic mutated genes in breast and colorectal cancers, and Tables S3, S4, and S5 from [11] for somatic mutated and CNV genes in pancreatic cancers.

All network diagrams were drawn with Cytoscape [97]. The functional enrichment analyses for pathways and GO annotations were based on binomial test. False discovery rates were calculated based on 1,000 permutations on all genes in the FI network.

### Enhanced experimental SkyPainter

Skypainter is a web application implemented in the Reactome web site for gene or protein over-representation analysis [23]. We augmented the function of the original Skypainter by adding predicted FI data. The enhanced Skypainter was implemented using the Google web toolkit [98].

## Abbreviations

CNV: copy number variation; EGFR: epidermal growth factor receptor; FI: functional interaction; GBM: glioblastoma multiforme; GO: Gene Ontology; HPRD: Human Protein Reference Database; NBC: naïve Bayes classifier; PPI: protein-protein interaction; TCGA: The Cancer Genome Atlas; TGF: transforming growth factor; TNC: tenascin-C.

## Authors' contributions

GW designed the study, constructed the FI network, did network analysis for cancer data sets, and drafted the manuscript. XF studied biological properties of the FI network. LS conceived of the study, participated in its design, and edited the manuscript. All authors have read and approved the final manuscript.

## Supplementary Material

Additional file 1 **Supplementary materials**. Click here for file

Additional file 2 **FI database mysql dump file**. Click here for file

Additional file 3 **Six FI files plus one read-me file explaining what these files are**. Click here for file
