# Supplementary material for: A human functional protein interaction network and its application to cancer data analysis
Source: Genome Biol. 2010 May 19;11(5):R53. doi: 10.1186/gb-2010-11-5-r53 (PMC2898064; doi:10.1186/gb-2010-11-5-r53)
Supplement: Additional file 1 — Supplementary materials. [file gb-2010-11-5-r53-S1.PDF]

A Human Functional Protein Interaction Network and Its Application to Cancer Data Analysis

Guanming Wu[1*], Xin Feng[2,3], and Lincoln Stein[1,2]

[1] Ontario Institute for Cancer Research, Toronto, ON Canada

[2] Cold Spring Harbor Laboratory, Cold Spring Harbor, NY USA

[3] State University of New York at Stony Brook, Stony Brook, NY USA

[*]To whom correspondence should be addressed.

Keywords: bioinformatics, databases, functional interactions, pathways, cancer, network clustering

Supplementary Materials

Figure S1: Venn diagrams showing feature contributions for FI prediction. Predicted FIs have been split into two parts: FIs having at least one PPI, and FIs having no PPI feature. A: Predicted FIs having at least on feature from one of five PPIs (human PPIs, Fly PPIs, Worm PPIs, Yeast PPIs, and PPIs from GeneWays). The number of predicted FIs having at least one PPI feature is 104747 (97% of total 111398 predicted FIs). B: Predicted FIs having no feature from one of five PPIs. The number of this subset of predicted FIs is 3581 (3% of predicted FIs). All FIs in this subset have at least one feature from one of the two gene coexpression data sets. *BP Sharing:* GO biological process term annotation sharing; *Gene Exp:* gene coexpression.

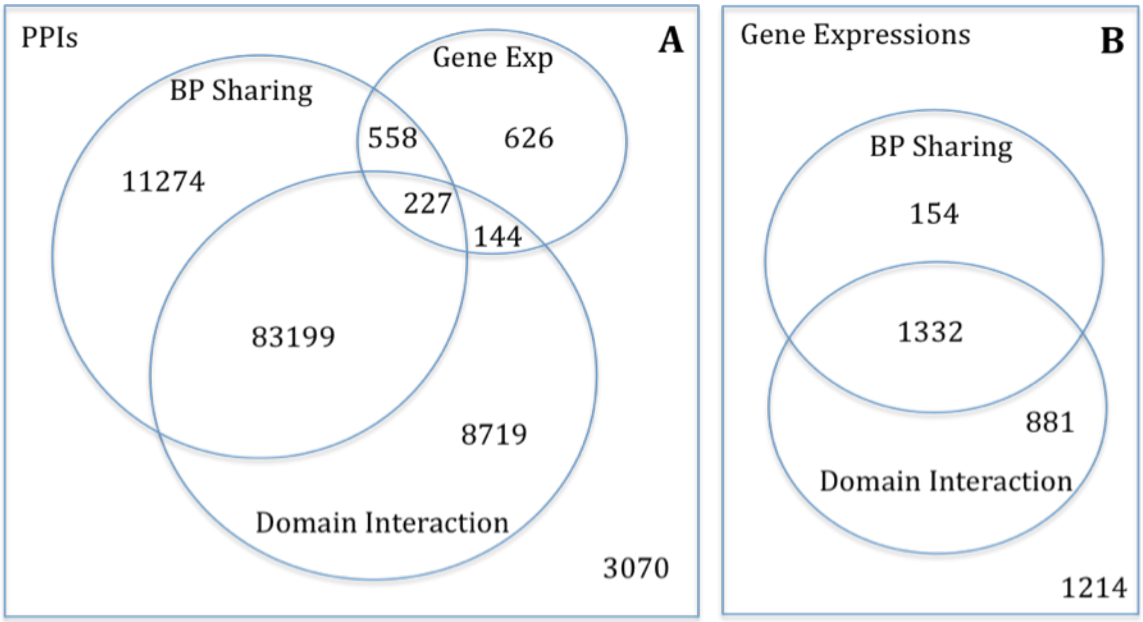

Figure S2: Overlay of functional interactions onto a human curated GBM pathway from the TCGA data set. Based on the FI network, more FIs may exist among original pathway components. However, in this diagram, only FIs between newly added proteins and pathway components are added. Newly added proteins are colored in light blue, while original components in grey. Newly added FIs are in blue, while original interactions in other colors. FIs extracted from pathways are in solid lines (e.g. PHLPP -> AKT1), while ones predicted based on NBC in dashed lines (e.g. KLF6 – TP53). Extracted FIs involved in activation, expression regulation, or catalysis are labeled with arrows, while FIs involved in inhibition labeled with "T". The original GBM pathway map in the Cytoscape format was downloaded from http://cbio.mskcc.org/cancergenomics/gbm/.

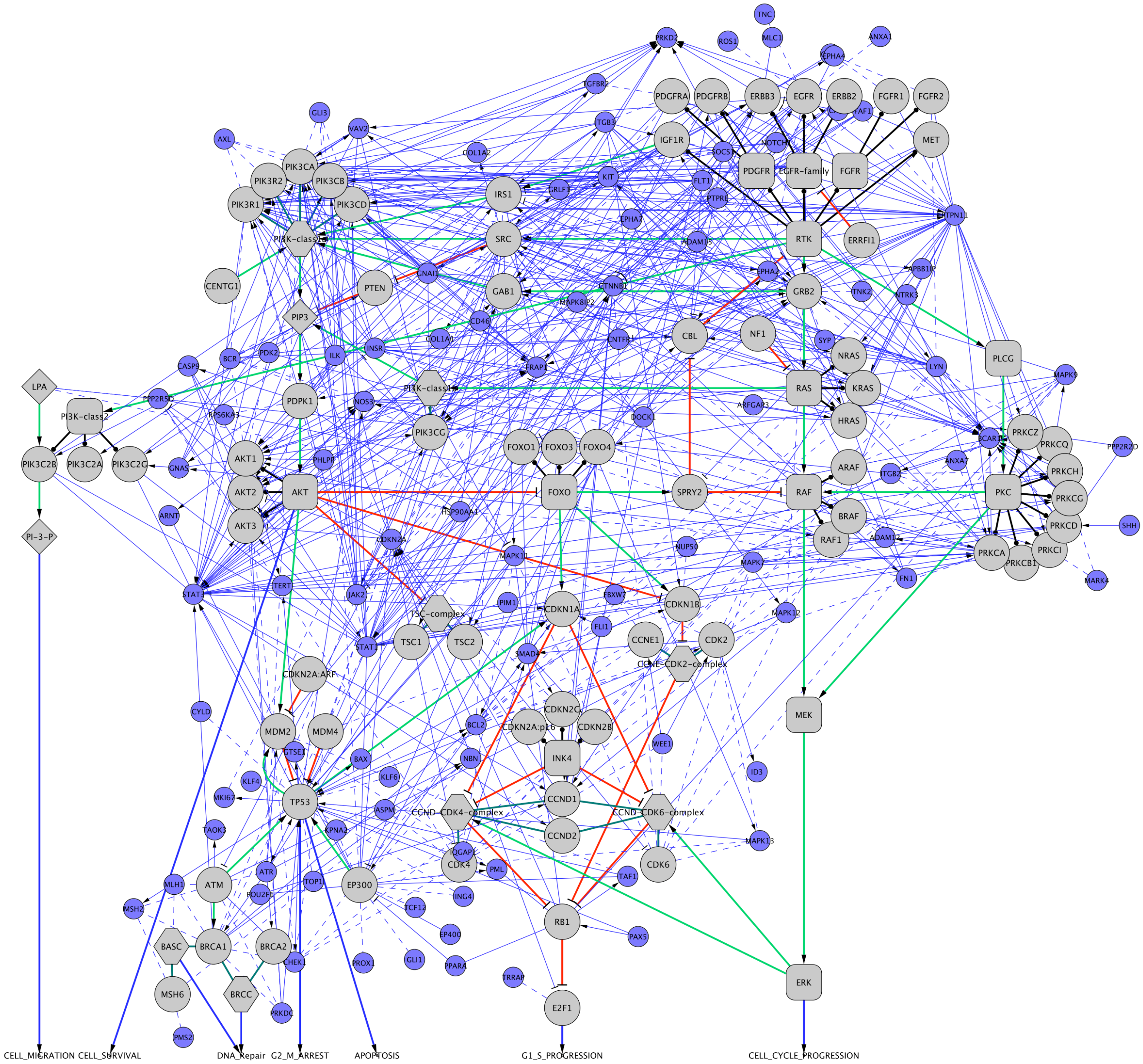

Figure S3: Edge-betweenness network clustering results for the altered genes from the Parsons data set. Gene nodes in different clusters are displayed in different colors. The node size is proportional to the number of samples bearing displayed altered genes.

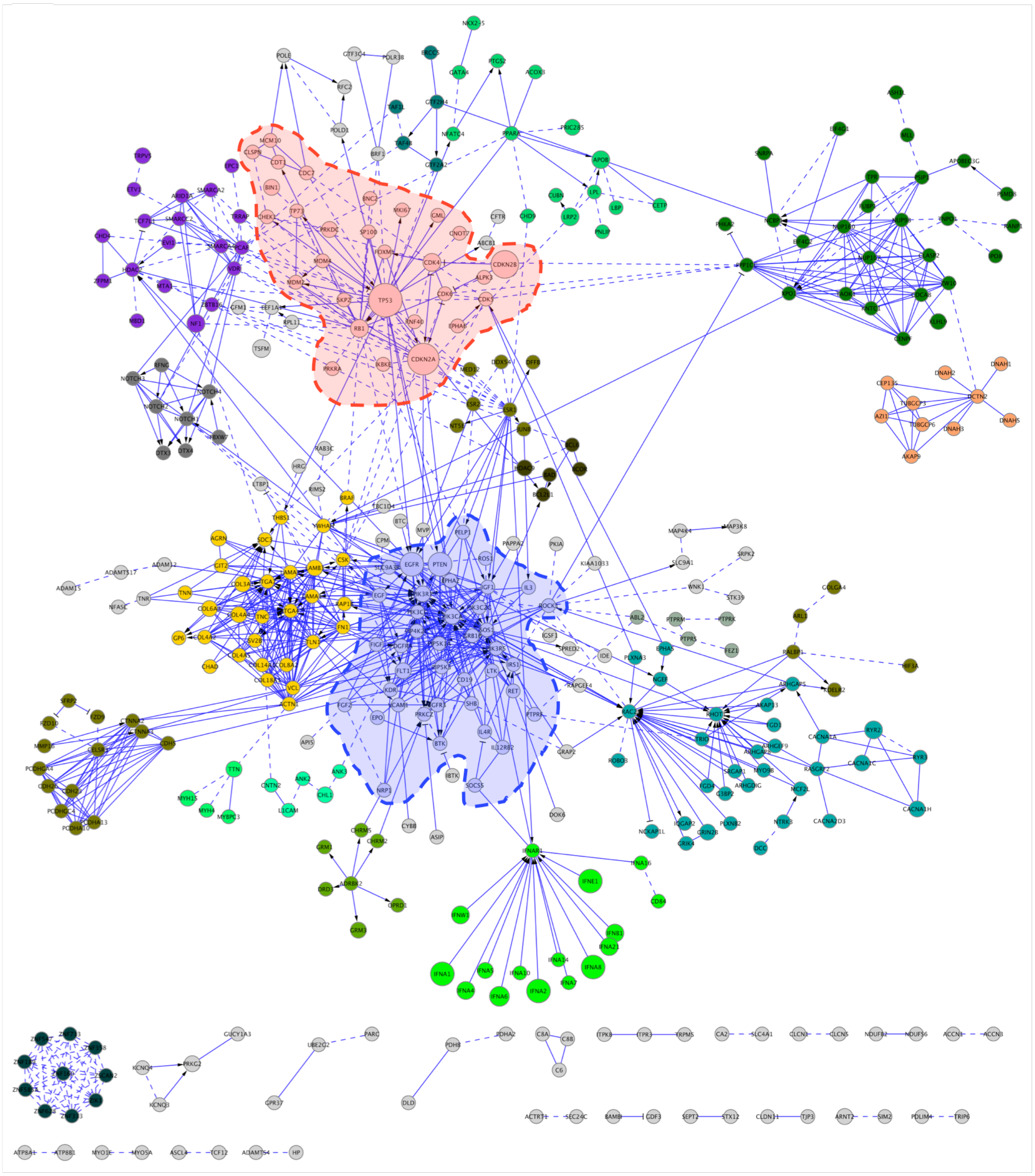

Figure S4. Edge-betweenness network clustering results for the mutated genes from the breast cancer data set. Gene nodes in different clusters are displayed in different colors. The node size is proportional to the number of samples bearing displayed mutated genes.

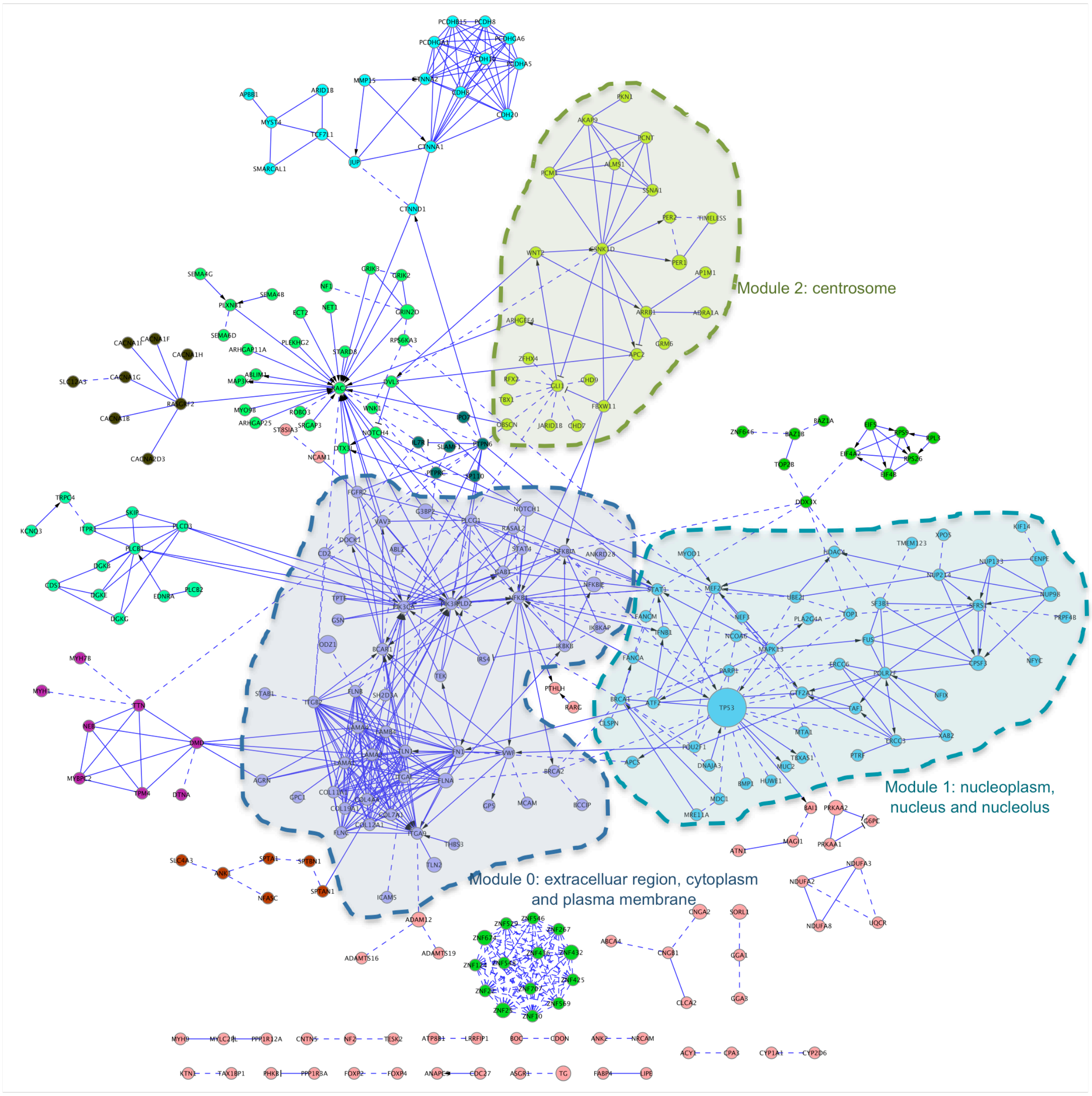

Figure S5: Edge-betweenness network clustering results for the mutated genes from the colorectal cancer data set. Gene nodes in different clusters are displayed in different colors. The node size is proportional to the number of samples bearing displayed mutated genes.

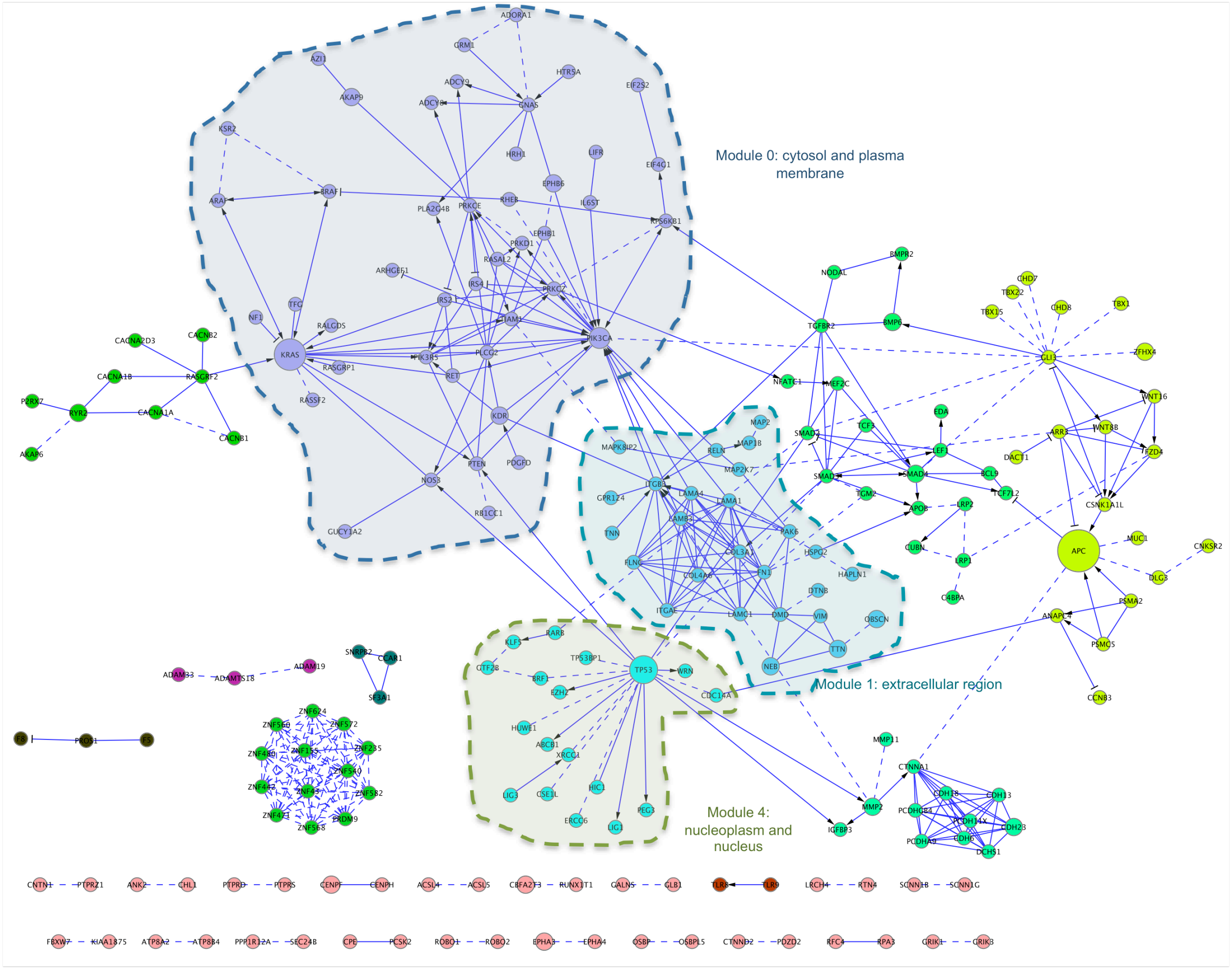

Figure S6: Sub-networks with pathways annotated for the breast cancer cluster from hierarchical clustering. Genes mutated in at least two cancer samples are in aqua, and linker genes used to connect cancer genes in brown. The node size is proportional to the number of samples bearing displayed mutated genes. Only two pathways are labeled in the diagram to keep image readable though many more pathways are significantly hit by the cluster.

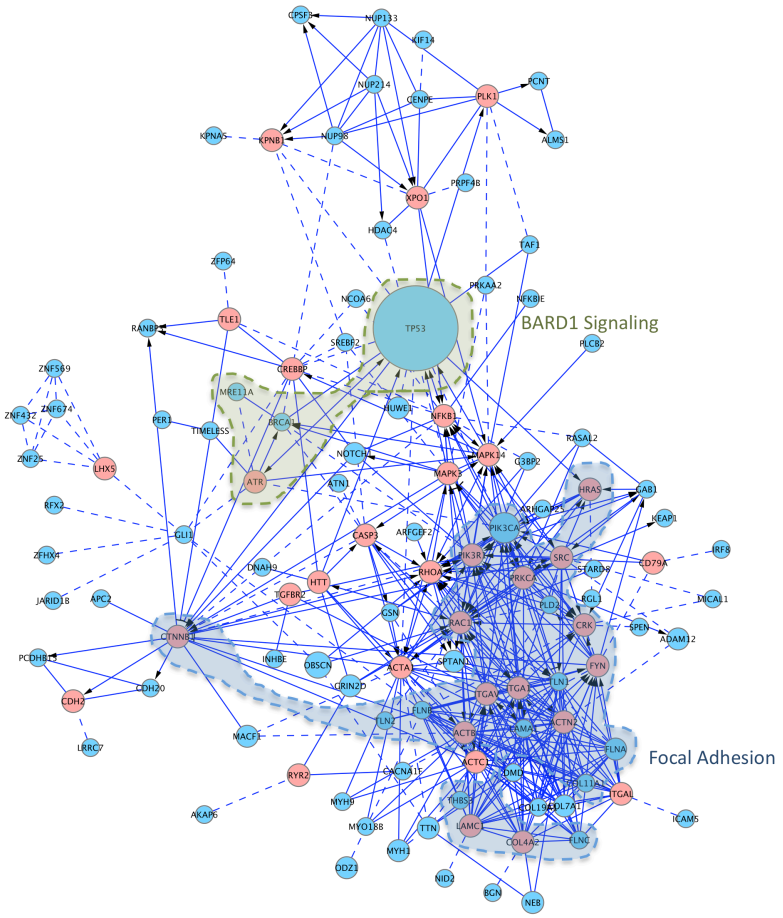

Figure S7: Sub-network with pathways annotated for the colorectal cancer cluster from hierarchical clustering. Three pathways are labeled in this diagram.

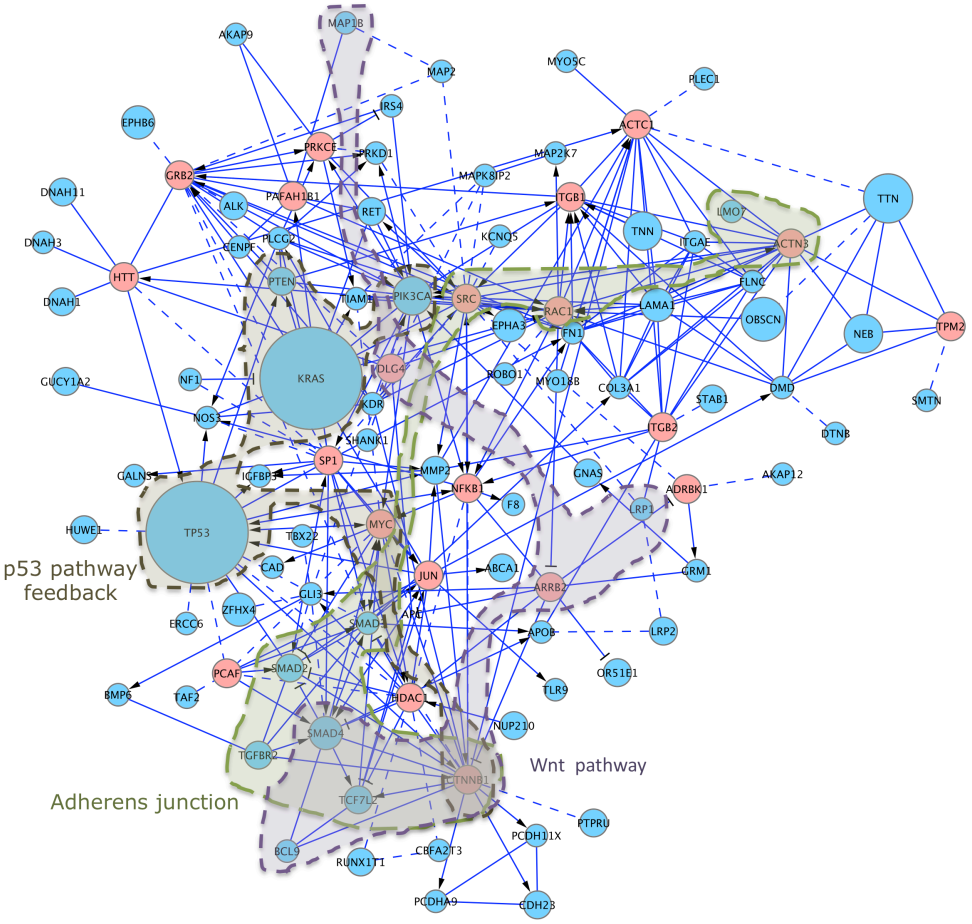

Figure S8: Edge-betweenness network clustering results for the sequence altered genes from the pancreatic cancer data set. Gene nodes in different clusters are displayed in different colors. The node size is proportional to the number of samples bearing displayed altered genes.

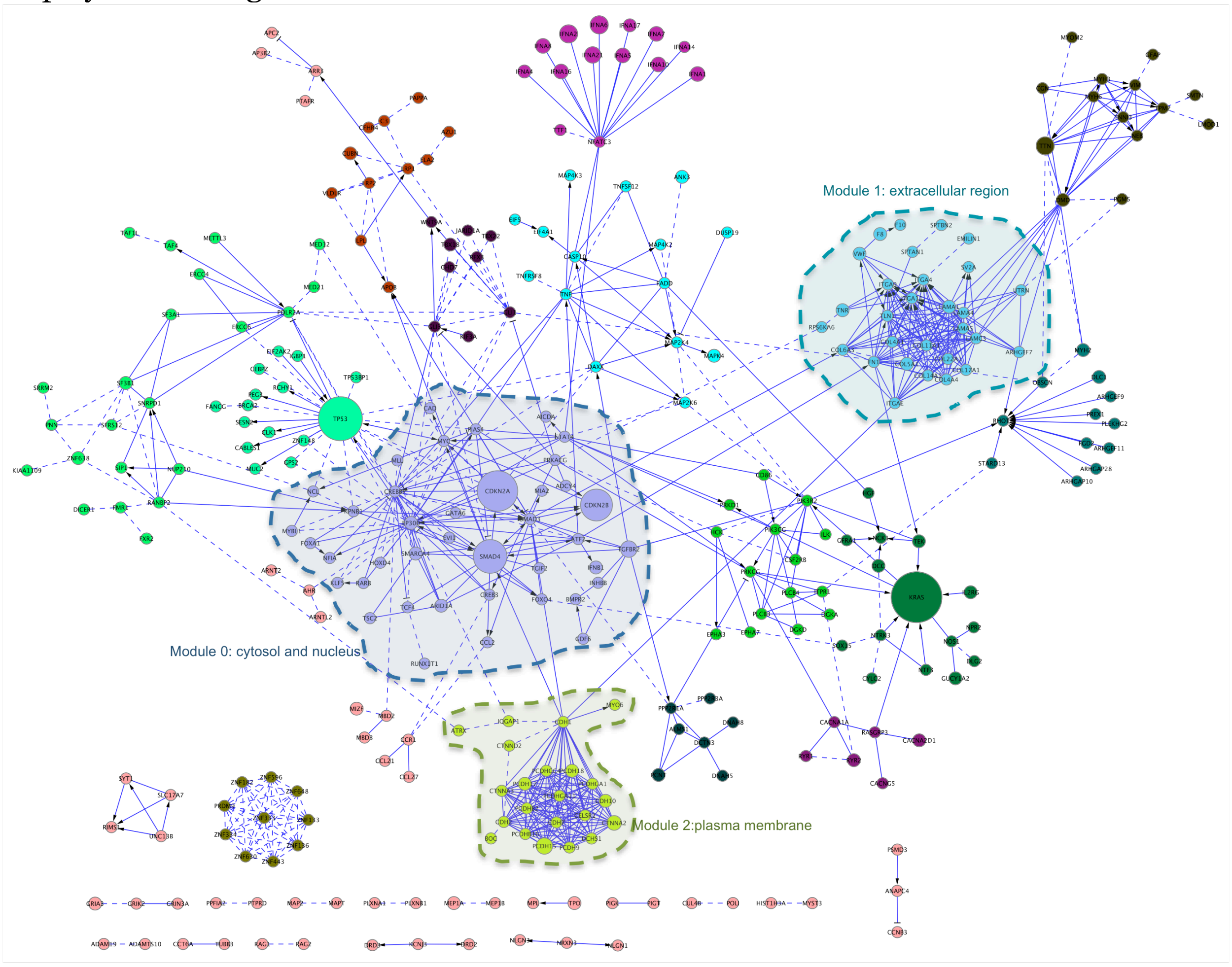

Figure S9: Sub-network with pathways annotated for the pancreatic cancer cluster from hierarchical clustering. Three pathways are labeled in this diagram.

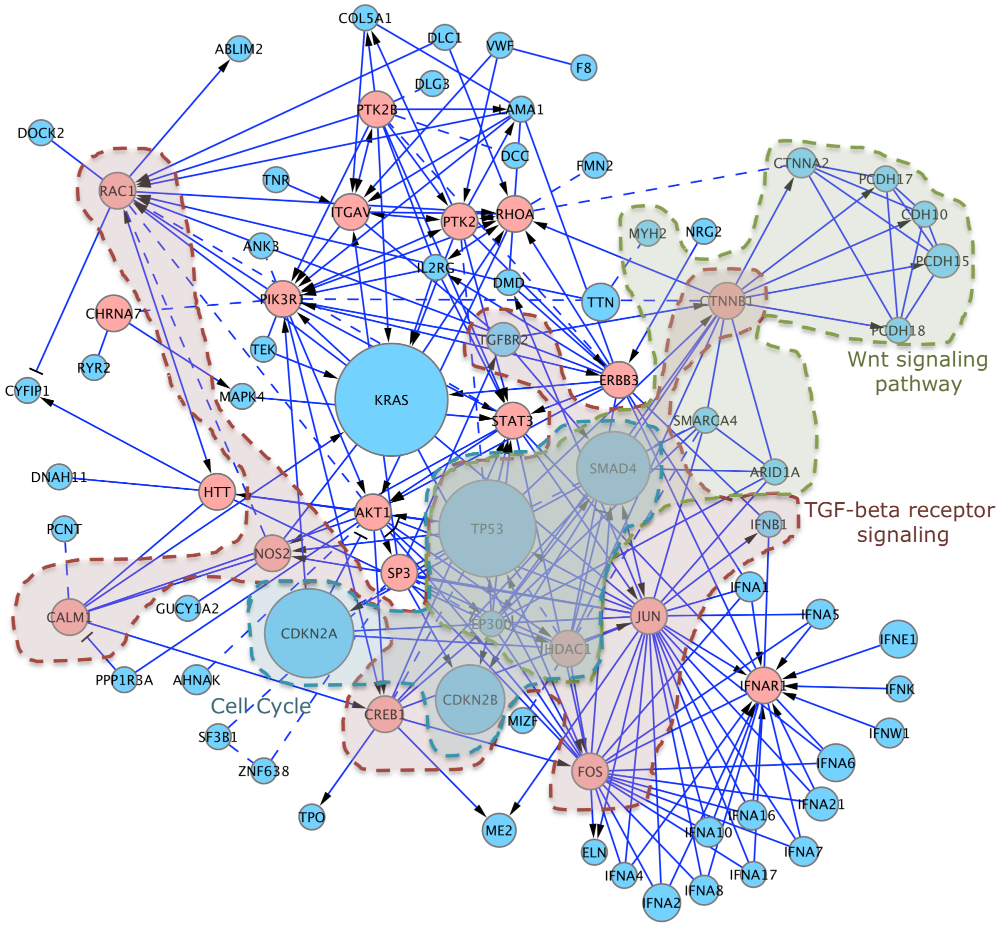

Table S1: The contribution of raw features to the FIs predicted by the NBC. The Feature Count column describes the starting number of interactions contained within each feature set. The Predicted FIs column describes the number of interactions that were contained within the feature set that contributed to the final FI set. This table excludes known interactions that are contained within the curated databases.

Feature	Feature Count	Predicted FIs	Percentage
Human PPIs	44819	14167	32%
Fly PPIs	937670	88372	9.4%
Worm PPIs	121837	4577	3.8%
Yeast PPIs	1898633	7881	0.42%
Domain Interaction	N/A	93775	N/A
Lee's Gene Expression	200680	4089	2.0%
Prieto's Gene Expression	12232	2593	21%
GO BP Sharing	N/A	98241	N/A
PPIs from GeneWays	46794	5505	12%

Table S2: Pathway and Gene Ontology (GO) cellular component annotations for two major network modules (Module 0 and Module 1) identified by edge-betweenness clustering of sequence-altered genes in the TCGA data set. P-values in the table are calculated based on binomial test and FDR values based on 1000 permutation tests. Only the first four pathways and GO terms are listed based on p-values.

Pathway Annotations:

Module	Pathway	P-Value	FDR	Altered Genes in Pathway
0	Sphingosine 1-phosphate (S1P) pathway(N)	8.88E-16	0.00E+00	JAK2, PDGFRB, PIK3R1, PRKCZ, IRS1, GNAI1, BCL2, PIK3CA, VAV2, PTPN11, BCAR1, FLT1, STAT3, CBL, LYN, STAT1, PRKCD, PTEN
0	EGF receptor signaling pathway(P)	1.22E-15	0.00E+00	KRAS, SPRY2, MAPK11, PIK3C2B, PRKCZ, NRAS, PRKCA, PIK3CA, EGFR, ERBB3, ERBB2, STAT3, PRKD2, CBL, MAPK9, STAT1
0	Signaling events mediated by Stem cell factor receptor (c-Kit)(N)	9.66E-15	0.00E+00	JAK2, PIK3C2B, PIK3R1, SOCS1, BCL2, PIK3CA, PTPN11, STAT3, KIT, CBL, LYN, STAT1, PTEN
0	IL2-mediated signaling events(N)	2.54E-14	0.00E+00	KRAS, MAPK11, PIK3R1, PRKCZ, SOCS1, IRS1, BCL2, NRAS, PRKCA, PIK3CA, PTPN11, FRAP1, STAT3, TERT, MAPK9, STAT1
1	Cell cycle(K)	2.15E-14	0.00E+00	CHEK1, EP300, PRKDC, MDM2, TP53, CDKN2A, CDKN2B, ATM, SMAD4, WEE1, CDKN2C, ATR, CDK4, RB1
1	Glypican 1 network(N)	2.13E-10	0.00E+00	FOXO4, CTNNB1, EP300, PRKDC, MDM2, TP53, KPNA2, CDKN2A, PML, CDKN2B, ATM, GATA3, SMAD4, FOXO3, CYLD, CDK4, POU2F1, TAOK3
1	p53 pathway(P)	2.67E-10	0.00E+00	GTSE1, EP300, MDM2, MYST4, TP53, CDKN2A, PML, ATM, ATR
1	Glypican pathway(N)	7.68E-10	0.00E+00	FOXO4, CTNNB1, EP300, PRKDC, MDM2, TP53, KPNA2, CDKN2A, PML, CDKN2B, ATM, GATA3, SMAD4, FOXO3, CYLD, CDK4, POU2F1, TAOK3

GO Cellular Component Annotations:

Module	GO Term	P-Value	FDR	Altered Genes with GO term annotated
0	cytosol	1.37E-06	0.00E+00	SPRY2, MAPK11, PIK3C2B, PIK3R1, BCL2, PRKCA, PIK3CA, ANXA7, PTPN11, FRAP1, NOS3, INSR, TSC1, FAF1, CBL, TSC2, PRKCD, PTEN
0	membrane fraction	2.95E-06	0.00E+00	ROS1, KRAS, MET, PRKCZ, BCL2, NRAS, PRKCA, BCAR1, TSC1, TSC2, FGFR1
0	caveola	6.58E-06	0.00E+00	JAK2, IRS1, NOS3, INSR
0	cytoplasm	2.66E-05	1.00E-03	JAK2, SPRY2, PIK3C2B, ILK, PIK3R1, NTRK3, PRKCZ, SOCS1, IRS1, PIM1, BCL2, PRKCA, A2M, PIK3CA, NF1, ANXA1, EGFR, PTPN11, BCAR1, MLC1, ERBB2, FRAP1, NOS3, PPP2R2D, STAT3, TSC1, FAF1, KIT, CBL, LYN, TSC2, DOCK1, STAT1, PRKCD, PTEN
1	nucleus	5.56E-13	0.00E+00	PPARA, ASPM, KLF6, FOXO4, ING4, TAF1, CTNNB1, CHEK1, PMS2, EP300, PRKDC, ID3, MLH1, TCF12, TOP1, MDM2, MYST4, TP53, MKI67, MSH2, KPNA2, CDKN2A, PML, ATM, GATA3, MYCN, SMAD4, TRRAP, BRCA2, PROX1, WEE1, FOXO3, NBN, CDKN2C, MSH6, KLF4, ARNT, ATR, CDK4, EP400, MDM4, RB1, PAX5, POU2F1
1	nucleoplasm	3.45E-11	0.00E+00	TAF1, CHEK1, PRKDC, TOP1, MDM2, TP53, KPNA2, PML, ATM, SMAD4, BRCA2, WEE1, NBN, ATR, CDK4, RB1
1	replication fork	2.74E-05	3.33E-04	CHEK1, TP53, NBN
1	transcription factor complex	3.36E-05	2.50E-04	CTNNB1, EP300, TCF12, TRRAP, CDK4, RB1

Table S3: Pathway and GO cellular component annotations for three major network modules (Modules 0, 1 and 2) identified by edge-betweenness clustering of mutated genes in breast cancers. P-values in the table are calculated based on binomial test and FDR values based on 1000 permutation tests. Only the first four pathways based on p-values and major GO terms are listed.

Pathway Annotations

Module	Pathway	P-value	FDR	Mutated Genes in Pathway
0	Integrin signalling pathway(P)	1.11E-16	0.00E+00	FLNA, COL4A4, FN1, PIK3R1, FLNB, ABL2, LAMB4, LAMA1, PIK3CA, TLN1, FLNC, BCAR1, COL7A1, COL11A1, COL19A1, COL12A1, LAMA2, DOCK1, LAMA4, ITGB2, ITGAL, ITGA9
0	Focal adhesion(K)	5.55E-16	0.00E+00	FLNA, THBS3, COL4A4, FN1, PIK3R1, FLNB, LAMB4, LAMA1, PIK3CA, TLN1, FLNC, BCAR1, VWF, COL11A1, LAMA2, DOCK1, LAMA4, TLN2, ITGA9
0	ECM-receptor interaction(K)	5.13E-12	0.00E+00	THBS3, COL4A4, FN1, AGRN, LAMB4, LAMA1, VWF, GP5, COL11A1, LAMA2, LAMA4, ITGA9
0	Integrin cell surface interactions(R)	1.50E-09	0.00E+00	COL4A4, FN1, LAMA1, TLN1, BCAR1, VWF, LAMA2, ITGB2, ITGAL, ITGA9
1	Transcription(R)	6.05E-07	0.00E+00	CPSF3, TAF1, POLR2F, ERCC3, SFRS1, GTF2A1, NFIX, POU2F1, PTRF
1	Oxidative stress response(P)	1.21E-06	0.00E+00	ATF2, MAPK13, FUS, PLA2G4A, MEF2C, STAT1
1	DNA Repair(R)	2.80E-06	0.00E+00	XAB2, ERCC6, BRCA1, MDC1, POLR2F, ERCC3, MRE11A
1	HIV Life Cycle(R)	1.05E-05	0.00E+00	TAF1, NUP214, POLR2F, ERCC3, NUP98, GTF2A1, NUP133
2	G2/M Transition(R)	2.88E-07	0.00E+00	AKAP9, PCNT, CSNK1D, PCM1, ALMS1, SSNA1
2	Circadian rhythm(K)	1.36E-05	0.00E+00	CSNK1D, PER1, PER2
2	Hedgehog signaling pathway(K)	4.47E-05	3.00E-03	WNT2, CSNK1D, FBXW11, GLI1
2	Circadian clock system(P)	3.77E-04	1.83E-02	PER1, PER2

GO Cellular Component Annotations

Module	GO Term	P-Value	FDR	Altered Genes with GO term annotated
0	extracellular region	5.45E-05	1.67E-03	FGFR2, THBS3, COL4A4, ODZ1, FN1, GPC1, AGRN, LAMB4, LAMA1, GSN, COL7A1, VWF, COL11A1, COL19A1, COL12A1, LAMA2, LAMA4
0	basement membrane	5.72E-05	1.25E-03	FN1, LAMB4, LAMA1, COL7A1
0	cytoplasm	4.87E-03	3.84E-02	FLNA, NOTCH1, PIK3R1, FLNB, IKBKAP, STAT4, ABL2, VAV3, PIK3CA, TLN1, MCAM, FLNC, G3BP2, NFKBIA, BCAR1, GAB1, GSN, BRCA2, VWF, IKBKB, PLCG1, STAB1, NFKBIE, DOCK1, NFKB1, ITGB2, TLN2
0	plasma membrane	2.36E-02	2.49E-01	FGFR2, FLNA, NOTCH1, GPC1, ICAM5, VAV3, TEK, PLD2, TLN1, MCAM, FLNC, VWF, PLCG1, GP5, STAB1, IRS4, ITGB2, ITGAL, TLN2
1	nucleoplasm	1.48E-10	0.00E+00	CPSF3, XAB2, ERCC6, TAF1, CLSPN, BRCA1, MDC1, TOP1, TP53, POLR2F, ERCC3, NUP98, MRE11A, GTF2A1, PTRF
1	nucleus	5.13E-10	0.00E+00	SF3B1, CPSF3, CENPE, XPO5, HDAC4, PRPF4B, XAB2, ERCC6, ATF2, FANCM, TAF1, CLSPN, BRCA1, MYOD1, FUS, MDC1, NUP214, TOP1, TP53, POLR2F, ERCC3, NUP98, KIF14, FANCA, SFRS1, MTA1, NFYC, MEF2C, MRE11A, GTF2A1, STAT1, HUWE1, PARP1, NUP133, NFIX, NCOA6, UBE2I, POU2F1, PTRF
1	nucleolus	4.11E-08	0.00E+00	ERCC6, TAF1, FUS, TOP1, TP53, POLR2F, MTA1, MRE11A, GTF2A1, STAT1, HUWE1, PARP1, NCOA6
2	pericentriolar material	2.63E-04	1.70E-02	AKAP9, PCM1
2	centrosome	4.55E-04	1.75E-02	AKAP9, PCNT, ALMS1, SSNA1

Table S4: Pathway and GO cellular component annotations for three major network modules (Modules 0, 1 and 4) identified by edge-betweenness clustering of mutated genes in colorectal cancers. P-values in the table are calculated based on binomial test and FDR values based on 1000 permutation tests. Only the first four pathways (2 pathways only for module 4) based on p-values and major GO terms are listed.

Pathway Annotations

Module	Pathway	P-value	FDR	Mutated Genes in Pathway
0	Non-small cell lung cancer(K)	8.34E-08	0.00E+00	KRAS, PIK3R5, ARAF, BRAF, PIK3CA, PLCG2
0	Endothelin signaling pathway(P)	2.71E-07	0.00E+00	NOS3, PRKCZ, PRKCE, PIK3CA, GNAS, RET, ADCY8
0	Signalling by NGF(R)	3.06E-07	0.00E+00	KRAS, RALGDS, ADCY9, PRKCE, BRAF, ARHGEF1, PIK3CA, IRS2, PTEN, ADCY8
0	Endothelins(N)	3.42E-07	0.00E+00	RHEB, ADCY9, PRKCZ, PRKCE, RPS6KB1, EIF4G1, PIK3CA, GNAS, PTEN, ADCY8
1	ECM-receptor interaction(K)	5.22E-13	0.00E+00	ITGB3, FN1, LAMC1, TNN, LAMB3, COL3A1, LAMA1, HSPG2, LAMA4, RELN
1	Focal adhesion(K)	6.32E-11	0.00E+00	ITGB3, FN1, LAMC1, PAK6, TNN, LAMB3, COL3A1, LAMA1, LAMA4, FLNC, RELN
1	Integrin signalling pathway(P)	3.47E-09	0.00E+00	ITGAE, ITGB3, FN1, LAMC1, LAMB3, COL3A1, LAMA1, COL4A6, LAMA4, FLNC
1	agrin in postsynaptic differentiation(B)	2.34E-08	0.00E+00	LAMC1, PAK6, DMD, LAMB3, LAMA1, LAMA4
4	DNA Repair(R)	8.60E-07	1.00E-03	XRCC1, LIG1, ERCC6, TP53BP1, LIG3
4	hypoxia and p53 in the cardiovascular system(B)	1.66E-05	4.00E-03	HIC1, TP53, ABCB1

GO Cellular Component Annotations

Module	GO Term	P-Value	FDR	Altered Genes with GO term annotated
0	plasma membrane	4.94E-04	5.00E-02	PRKD1, KRAS, RHEB, IL6ST, NOS3, PRKCZ, HRH1, GRM1, IRS4, RASGRP1, ARHGEF1, HTR5A, PLCG2, IRS2, TIAM1, LIFR, RET, ADORA1, EPHB1, ADCY8
0	cytosol	7.41E-04	4.20E-02	PLA2G4B, PRKD1, RHEB, RALGDS, NOS3, RPS6KB1, EIF2S2, ARHGEF1, EIF4G1, PIK3CA, PTEN
0	membrane fraction	2.46E-03	7.80E-02	KRAS, PRKCZ, PRKCE, RASGRP1, GNAS, ADCY8
1	extracellular region	1.75E-04	7.00E-03	FN1, LAMC1, HAPLN1, LAMB3, COL3A1, LAMA1, COL4A6, HSPG2, LAMA4, RELN
4	nucleoplasm	1.53E-07	0.00E+00	GTF2B, WRN, XRCC1, LIG1, ERCC6, TP53, TP53BP1, LIG3
4	nucleus	1.04E-06	0.00E+00	RARB, HIC1, GTF2B, WRN, XRCC1, LIG1, EZH2, ERCC6, BRF1, PEG3, KLF5, TP53, HUWE1, TP53BP1, LIG3, CDC14A, CSE1L

Table S5: Clustering of sequence altered genes occurring in two or more samples from other cancer types in the FI network. Genes for breast and colorectal cancers are collected from somatic mutations only, while genes for pancreatic cancers from both somatic mutations and CNVs.

Cancer Type	Cancer Genes	Genes in FI network (%)	Genes in Cluster	Percentage of Genes in Cluster (P-value via random permutation)
**Breast**	197	113 (57%)	82	73% (0.003)
**Colorectal**	183	113 (62%)	80	71% (<0.001)
**Pancreatic**	132	79 (60%)	61	77% (<0.001)

Table S6: Average shortest distance for cancer clusters from other cancer types. The values in the cancer genes column are from the cancer clusters, while ones in the two permutation columns from permutation tests.

Cancer Type	Cancer Genes	Random Permutation (pvalue)	Degree-based Permutation (pvalue)
**Breast**	3.02	3.82 (< 0.001)	3.22 (0.001)
**Colorectal**	2.82	3.82 (< 0.001)	3.18 (<0.001)
**Pancreatic**	2.82	3.82 (< 0.001)	3.29 (<0.001)

Table S7: Pathway and GO cellular component annotations for three major network modules (Modules 0, 1 and 2) identified by edge-betweenness clustering of mutated and CNV genes in pancreatic cancers. P-values in the table are calculated based on binomial test and FDR values based on 1000 permutation tests. Only the first four pathways based on p-values and major GO terms are listed.

Pathway Annotations

Module	Pathway	P-value	FDR	Mutated Genes in Pathway
0	IFN-gamma pathway(N)	1.64E-10	0.00E+00	SMAD4, ATF2, FOXO4, MYBL1, MYC, EP300, TSC2, IFNB1, TGIF2, KPNB1, TGFBR2, AICDA, SMAD3, PIAS4, CDKN2B, CREBBP
0	Regulation of cytoplasmic and nuclear	7.28E-10	0.00E+00	SMAD4, ATF2, FOXO4, MYC, EP300, TSC2, IFNB1, TGIF2, KPNB1, TGFBR2, SMAD3, PIAS4, CDKN2B,

	SMAD2/3 signaling(N)			CREBBP
0	Regulation of nuclear SMAD2/3 signaling(N)	7.28E-10	0.00E+00	SMAD4, ATF2, FOXO4, MYC, EP300, TSC2, IFNB1, TGIF2, KPNB1, TGFBR2, SMAD3, PIAS4, CDKN2B, CREBBP
0	TGF-beta receptor signaling(N)	7.28E-10	0.00E+00	SMAD4, ATF2, FOXO4, MYC, EP300, TSC2, IFNB1, TGIF2, KPNB1, TGFBR2, SMAD3, PIAS4, CDKN2B, CREBBP
1	Focal adhesion(K)	1.11E-16	0.00E+00	COL4A4, FN1, ITGA4, VWF, COL11A1, ITGA11, COL5A1, COL4A1, LAMA5, COL6A3, LAMC3, LAMA1, LAMA4, TLN1, ITGA9, TNR
1	Integrin signalling pathway(P)	1.11E-16	0.00E+00	ITGAE, COL17A1, COL4A4, COL14A1, FN1, ITGA4, COL11A1, ITGA11, COL5A1, COL4A1, LAMA5, COL6A3, COL22A1, LAMC3, LAMA1, LAMA4, TLN1, ITGA9
1	ECM-receptor interaction(K)	1.11E-16	0.00E+00	COL4A4, FN1, ITGA4, VWF, COL11A1, ITGA11, COL5A1, COL4A1, LAMA5, COL6A3, LAMC3, LAMA1, SV2A, LAMA4, ITGA9, TNR
1	Integrin cell surface interactions(R)	1.11E-15	0.00E+00	ITGAE, COL4A4, FN1, ITGA4, VWF, ITGA11, COL4A1, LAMA5, LAMC3, LAMA1, TLN1, ITGA9
2	Wnt signaling pathway(P)	1.11E-16	0.00E+00	PCDH17, PCDH9, PCDHGA11, CDH10, PCDH18, PCDHB16, CELSR1, PCDH15, CTNNA3, PCDHGA1, CDH1, CTNNA2, PCDHGC4, CDH2, CDH7, DCHS1, PCDHB2
2	Cadherin signaling pathway(P)	1.11E-16	0.00E+00	PCDH17, PCDH9, PCDHGA11, CDH10, PCDH18, PCDHB16, CELSR1, PCDH15, PCDHGA1, CDH1, CTNNA2, PCDHGC4, CDH2, CDH7, DCHS1, PCDHB2
2	Adherens junction(K)	1.10E-04	3.33E-04	IQGAP1, CTNNA3, CDH1, CTNNA2
2	E-cadherin signaling in the nascent adherens junction(N)	5.60E-04	1.83E-02	IQGAP1, CDH1, MYO6

GO Cellular Component Annotations

Module	GO Term	P-Value	FDR	Altered Genes with GO term annotated
0	nucleus	5.97E-06	0.00E+00	CREB3, SMAD4, ATF2, MLL, FOXO4, KLF5, EVI1, HOXD4, MYBL1, CAD, MYC, RARB, EP300, STAT4, TSC2, NCL, NFIA, TGIF2, GATA6, KPNB1, RUNX1T1, ARID1A, SMARCA4, SMAD3, CDKN2A, FOXA1, PIAS4, TCF4, CREBBP
0	cytosol	3.96E-03	1.03E-01	CREB3, SMAD4, ATF2, FOXO4, CAD, TSC2, KPNB1, TGFBR2, SMAD3
1	extracellular region	5.65E-11	0.00E+00	COL17A1, F8, COL4A4, COL14A1, FN1, VWF, COL11A1, COL5A1, COL4A1, LAMA5, COL6A3, EMILIN1, COL22A1, LAMC3, LAMA1, F10, LAMA4, TNR
1	collagen	8.63E-09	0.00E+00	COL4A4, COL14A1, COL11A1, COL5A1, COL4A1
1	basement membrane	8.05E-08	0.00E+00	COL17A1, FN1, LAMA5, LAMC3, LAMA1
2	plasma membrane	4.54E-09	0.00E+00	IQGAP1, PCDH17, PCDH9, PCDHGA11, CDH10, PCDH18, CELSR1, PCDH15, PCDHGA1, BOC, CDH1, CTNNA2, PCDHGC4, CDH2, CDH7, DCHS1, MYO6, PCDHB2
2	integral to membrane	2.62E-06	0.00E+00	PCDH17, PCDH9, PCDHGA11, CDH10, PCDH18, PCDHB16, PCDH15, PCDHGA1, BOC, CDH1, PCDHGC4, CDH2, CDH7, DCHS1
